# Tumor endothelial cell up-regulation of IDO1 is an immunosuppressive feed-back mechanism that reduces the response to CD40-stimulating immunotherapy

**DOI:** 10.1080/2162402X.2020.1730538

**Published:** 2020-03-09

**Authors:** Maria Georganaki, Mohanraj Ramachandran, Sander Tuit, Nicolás Gonzalo Núñez, Alexandros Karampatzakis, Grammatiki Fotaki, Luuk van Hooren, Hua Huang, Roberta Lugano, Thomas Ulas, Aura Kaunisto, Eric C Holland, Peter Ellmark, Sara M Mangsbo, Joachim Schultze, Magnus Essand, Sonia Tugues, Anna Dimberg

**Affiliations:** a Department of Immunology, Genetics and Pathology, Science for Life Laboratory, The Rudbeck Laboratory, Uppsala University, Uppsala, Sweden; b Genomics & Immunoregulation, Life and Medical Science Institute, University of Bonn, Bonn, Germany; c Institute of Experimental Immunology, University of Zurich, Zurich, Switzerland; d Alligator Bioscience, Medicon Village, Lund, Sweden; e Division of Human Biology, Fred Hutchinson Cancer Research Center, Seattle, WA, USA; f Department of Pharmaceutical Biosciences, Science for Life Laboratory, Uppsala University, Uppsala, Sweden; g Platform for Single Cell Genomics and Epigenomics, The German Center for Neurodegenerative Diseases (DZNE) and the University of Bonn, Bonn, Germany

**Keywords:** IDO1, tumor endothelial cells, CD40, immunotherapy, melanoma

## Abstract

CD40-stimulating immunotherapy can elicit potent anti-tumor responses by activating dendritic cells and enhancing T-cell priming. Tumor vessels orchestrate T-cell recruitment during immune response, but the effect of CD40-stimulating immunotherapy on tumor endothelial cells has not been evaluated. Here, we have investigated how tumor endothelial cells transcriptionally respond to CD40-stimulating immunotherapy by isolating tumor endothelial cells from agonistic CD40 mAb- or isotype-treated mice bearing B16-F10 melanoma, and performing RNA-sequencing. Gene set enrichment analysis revealed that agonistic CD40 mAb therapy increased interferon (IFN)-related responses in tumor endothelial cells, including up-regulation of the immunosuppressive enzyme Indoleamine 2, 3-Dioxygenase 1 (IDO1). IDO1 was predominantly expressed in endothelial cells within the tumor microenvironment, and its expression in tumor endothelium was positively correlated to T-cell infiltration and to increased intratumoral expression of IFNγ. *In vitro*, endothelial cells up-regulated IDO1 in response to T-cell-derived IFNγ, but not in response to CD40-stimulation. Combining agonistic CD40 mAb therapy with the IDO1 inhibitor epacadostat delayed tumor growth in B16-F10 melanoma, associated with increased activation of tumor-infiltrating T-cells. Hereby, we show that the tumor endothelial cells up-regulate IDO1 upon CD40-stimulating immunotherapy in response to increased IFNγ-secretion by T-cells, revealing a novel immunosuppressive feedback mechanism whereby tumor vessels limit T-cell activation.

## Introduction

Antibodies stimulating CD40 signaling (agonistic CD40 mAb) show promising results with manageable toxicities in several clinical trials.^1,2^ CD40-activating immunotherapy primarily occurs through stimulation of dendritic cells (DCs) that subsequently prime T-cells via tumor antigen presentation. CD40 stimulation of DCs enhances co-stimulation, leading to expansion of tumor-specific T-cells and further activation of the antigen-presentation machinery in response to the produced IFNγ.^3–6^ The effect of CD40 receptor engagement is context dependent. In several tumor models, CD8^+^ T-cells are required for agonistic CD40 mAb efficacy.^7,8^ However, in pancreatic ductal adenocarcinoma, the anti-tumor effects of agonistic CD40 mAb are mediated via direct macrophage activation.^9^ CD40 is expressed by a wide variety of immune cells, and is also expressed by non-hematopoietic cells including endothelial cells.^10^

The role of endothelial cells in modulating the response to CD40-stimulating immunotherapy is not clear. CD40-stimulation induces endothelial activation *in vitro*, suggesting that this may enhance leukocyte adhesion and increase the therapeutic efficacy.^11–14^ However, CD40-stimulation can induce production of vascular endothelial growth factor (VEGF) in some models, promoting angiogenesis and increasing tumor growth *in vivo*.^15–17^ Increased pro-angiogenic signaling can interfere with expression of adhesion molecules required for T-cell adhesion to vessel walls and trans-endothelial migration.^18–21^ Decreasing VEGF-signaling through co-treatment with sunitinib can enhance the efficacy of agonistic CD40 mAb immunotherapy, associated with increased endothelial activation and enhanced CD8^+^ T-cell recruitment and activation.^22^

In addition to regulating T-cell recruitment, endothelial cells can express molecules that reduce T-cell activation or viability, including Programmed Cell Death-Ligand (PD-L)1, PD-L2, Fas ligand (FASL), TNF-related apoptosis-inducing ligand (TRAIL) and Indoleamine 2, 3-Dioxygenase 1 (IDO1).^23–25^ IDO1 is a rate-limiting enzyme for tryptophan (Trp) metabolism that restricts T-cell proliferation through Trp depletion, leading to growth arrest in the G1 phase of the cell cycle. Additionally, metabolism of Trp leads to accumulation of its catabolites that induce T-cell apoptosis and promote Tregs through the activation of aryl hydrocarbon receptor (AhR).^26,27^ IDO1 is differentially expressed in human tumors, and can be expressed by several different cell types within the tumor tissue.^28,29^ Most murine tumors do not constitutively express IDO1, but it can be induced by IFNγ secretion in the tumor microenvironment.^28,29^

Here we have identified changes in endothelial gene expression associated with agonistic CD40 mAb immunotherapy through isolation of tumor endothelial cells from B16-F10 melanoma followed by RNA-sequencing. We found that agonistic CD40 mAb treatment increased immunosuppression in tumor endothelial cells characterized by up-regulation of IDO1. Tumor endothelial cell expression of IDO1 was positively correlated with T-cell infiltration and IFNγ expression in the tumor. Consistent with this, IDO1 was increased in cultured endothelial cells in response to T-cell-derived IFNγ. Combining agonistic CD40 mAb therapy with the IDO1-inhibitor epacadostat resulted in increased T-cell activation, delayed tumor growth and increased survival in B16-F10 tumors. Our results demonstrate that tumor endothelial cells contribute to immunosuppressive feed-back loops in response to agonistic CD40 mAb therapy, which restrict the response to immunotherapy through IFNγ-driven expression of IDO1 that inhibits T-cell activation.

## Results

### Interferon-driven induction of genes in tumor endothelial cells in response to anti-CD40 treatment

To investigate endothelial responses to CD40-stimulation, we employed a panel of human (HDBEC and HUVEC) and murine endothelial cells (bEND3 and MS1) with varying basal levels of CD40 expression (Supplementary Fig S1A). To stimulate CD40 on endothelial cells, we used MegaCD40L, an artificial protein in which two trimeric CD40 ligands are linked through the collagen domain of adiponectin which mimics membrane-assisted CD40L aggregation. In addition, we stimulated mouse endothelial cells with the agonistic CD40 mAb FGK4.5. We did not observe induction of endothelial activation in response to CD40-stimulation, as analyzed by surface expression of the adhesion molecules ICAM-1, VCAM-1 and E-selectin, in either human or murine endothelial cells (Supplementary Fig S1B-K). In contrast, MegaCD40L and agonistic CD40 mAb induced expression of the activation markers CD86 and HLA-DR in human monocyte-derived DCs (moDCs) and CD86 in the dendritic cell (DC) D1 cell line, confirming that these reagents are efficient CD40 agonists (Supplementary Fig S1 L-N).

**Figure 1.**
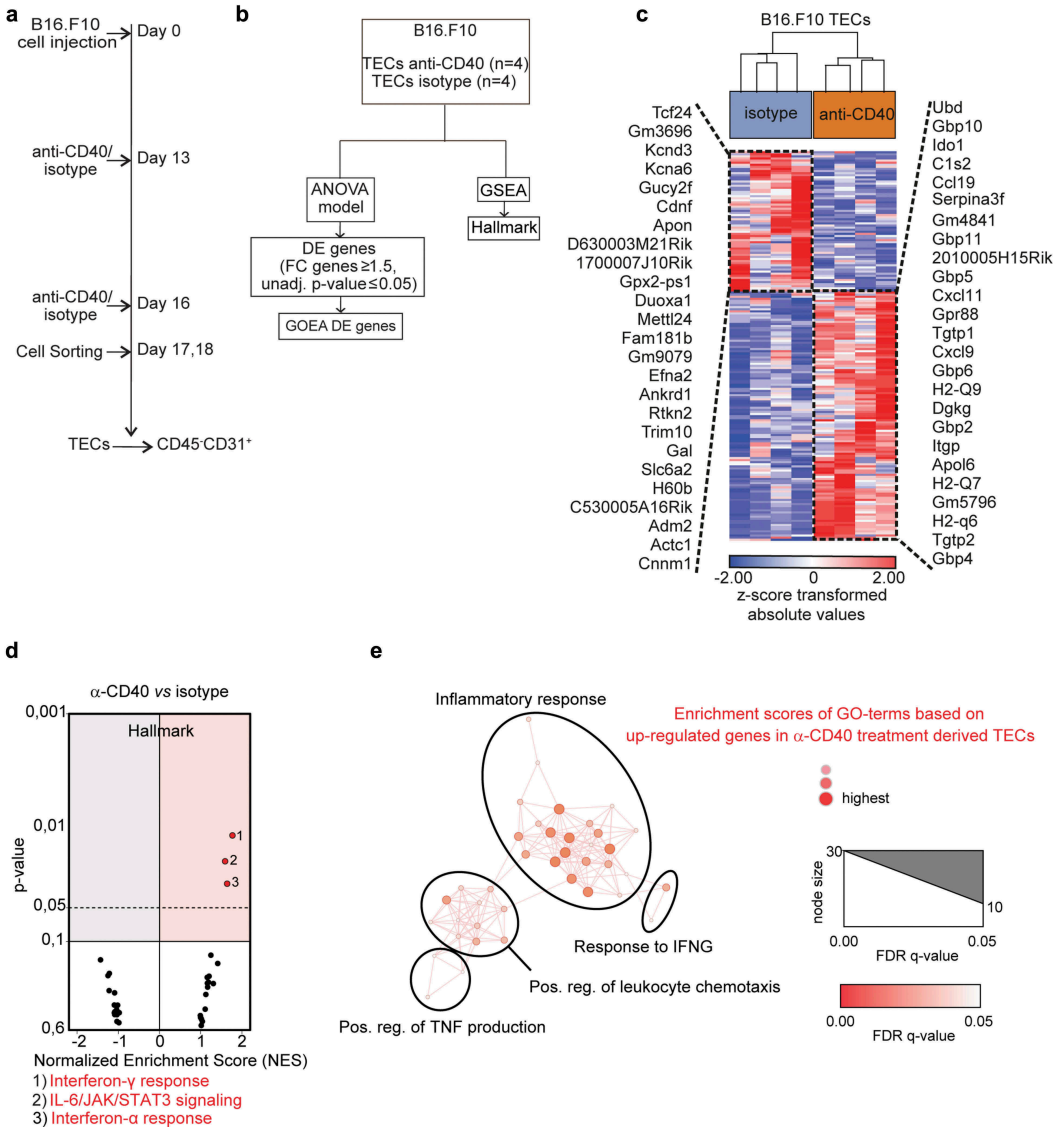
Transcriptome analysis of tumor endothelial cells from agonistic CD40 mAb or isotype control treated B16-F10 tumors: (a-b) Schemes outlining experimental setup (a) and bio informatic analysis (b) of tumor endothelial cells (TECs). (c) Hierarchical clustering of differentially expressed (DE) genes (119 upregulated; 67 downregulated) between agonistic CD40 mAb (n = 4 tumors) and isotype control treated (n=4 tumors) B16-F10-derived tumor endothelial cells (TECs) (FC 1.5; unadjusted p<≤0.05). The top 25 upregulated and downregulated genes are indicated. (d) Volcano plots of normalized enrichment scores (NES) and enrichment p-values in B16-F10 tumor endothelial cells based on GSEA using Hallmark pathway gene sets. Red circles (NES ≥ 1; p value ≤ 0.1) show gene sets positively enriched. (e) Network visualization of GOEA based on DE genes in tumor endothelial cells in response to anti-CD40 treatment in B16-F10 tumors using BiNGO and Enrichment Map. Red nodes represent the enriched GO-terms, node size and color represent corresponding FDR-adjusted enrichment p-values (p-value ≤ 0.05).

Tumor endothelial cells adapt to their microenvironment, and respond to changes in the microenvironment that can be induced by cancer therapy. Therefore, we analyzed the effect of agonistic CD40 mAb immunotherapy on tumor endothelial cells *in vivo*. We isolated tumor endothelial cells from B16-F10 melanoma treated with either agonistic CD40 mAb (FGK4.5) or isotype control and generated genome-wide transcriptome data by RNA-sequencing (Figure 1a). Transcriptional changes induced by agonistic CD40 mAb were determined through bioinformatic analysis (Figure 1b). We employed a two-way ANOVA model to assess and visualize the degree of transcriptional variance between agonistic CD40 mAb and isotype control treated tumor endothelial cells, and applied hierarchical clustering to visualize differentially expressed genes as a heatmap (Supplementary Table S3, Figure 1c).

**Figure 2.**
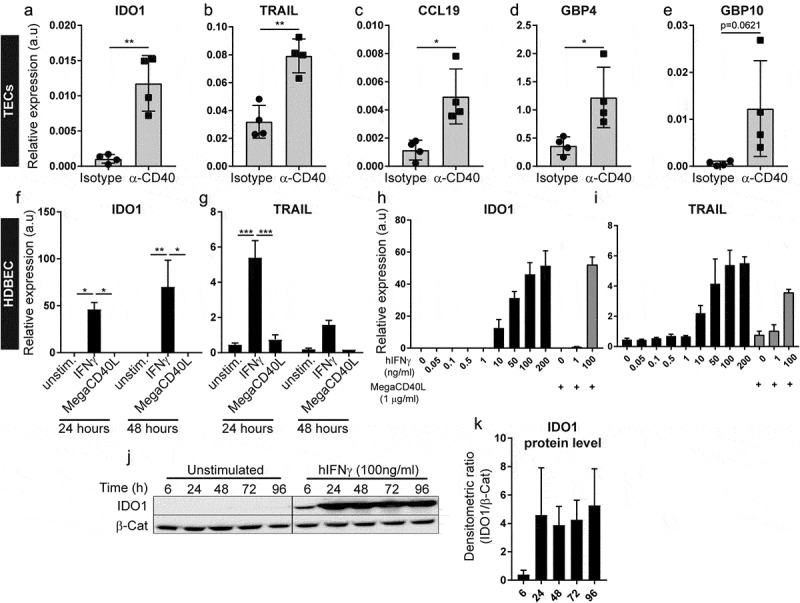
Validation of selected genes found to be differentially expressed in tumor endothelial cells in response to CD40-stimulating immunotherapy: (a-e) Quantitative PCR (qPCR) detection of genes previously identified to be differentially expressed by RNA-sequencing (a) IDO1. (b) TRAIL, (c) CCL 19, (d) GBP4, (e) GBP10, in TECs derived from B16-F10 tumors treated with agonistic CD40 mAb (n = 4) or isotype control (n = 4). Graphs depict relative target gene expression compared to HPRT housekeeping gene expression. (f-g) qPCR detection of IDO1 (f) and TRAIL (g) expression levels in HDBEC treated with human recombinant IFNγ (100 ng/ml) or MegaCD40L (1µg/ml) for 24 and 48 hours (mean values show relative expression compared to HPRT from biological duplicates). (H-1) qPCR detection of IDO1 (h) and TRAIL (i) expression levels in HDBEC treated with increasing doses (0-200ng/ml) of human recombinant IFNγ (100 ng/ml) and MegaCD40L (1µg/ml) for 18 hours. (j-k) Western blot of lDOI protein in HDBEC treated with 100 ng/ml human recombinant IFNγ for up to 4 days (i) and analysis of IDO1 relative to β-catenin protein levels (k) from 3 independent experiments. Mean, SD, *p<0.05, **p<0.01, unpaired Student's t-test.

Biological pathways induced in tumor endothelial cells upon agonistic CD40 mAb treatment were explored by Gene Set Enrichment Analysis (GSEA) using the hallmark-curated pathway gene sets (Supplementary Table S4).^30^ Strikingly, tumor endothelial cells from agonistic CD40 mAb treated B16-F10 tumors mainly upregulated inflammation-related hallmark pathways related to interferon (IFN)γ and IFNα response and IL-6/JAK/STAT3 signaling (Figure 1d). To predict the biological outcome of the transcriptional response observed in tumor endothelial cells, we conducted GOEA and visualized enriched GO terms using BiNGO and Enrichment Map (Figure 1e). We found that tumor endothelial cells from agonistic CD40 mAb treated tumors upregulated biological processes such as “inflammatory response”, “response to IFNγ”, “positive regulation of leukocyte chemotaxis” and “positive regulation of TNF production”. Notably, activation of IFN signaling in response to agonistic CD40 mAb treatment was also associated with expression of genes previously suggested to be part of a tumor endothelial barrier that limits T-cell infiltration, activation and viability, including IDO1 and TNFSF10 (TRAIL) (Supplementary Table S3).^24^ To validate our results, we performed quantitative PCR (qPCR) analysis of gene expression for selected DE candidate genes in the original samples used for RNA-sequencing from the B16.F10 tumors. IDO1, TRAIL, CCL19 and GBP4 were significantly upregulated in agonistic CD40 mAb treated samples compared to isotype control treated, whereas GBP10 expression was highly variable (Figure 2a–e). Taken together, our results indicate that agonistic CD40 mAb treatment of mice bearing B16-F10 melanoma is associated with increased IFN signaling in tumor endothelial cells, up-regulation of inflammatory pathways and increased expression of molecules associated with increased endothelial barrier function and immunosuppression such as TRAIL and IDO1.

### IFNγ stimulation of endothelial cells induces expression of IDO1 and TRAIL

The bioinformatic analysis of differential gene expression in response to agonistic CD40 mAb therapy indicated that enhanced IFN-signaling in the tumor microenvironment underlies the transcriptional response of tumor endothelial cells. Interferon signaling promotes anti-tumor immune response, but can also induce immunosuppressive mechanisms in cancer.^31^ To identify mechanisms leading to enhanced IDO1 and TRAIL expression in endothelial cells after CD40-stimulating immunotherapy, we analyzed gene regulation in human umbilical vein endothelial cells (HUVEC) and human dermal blood endothelial cells (HDBEC) *in vitro*. Interestingly, whereas stimulation with the CD40 agonist MegaCD40L did not affect expression of either IDO1 or TRAIL, human recombinant IFNγ treatment elevated IDO1 and TRAIL mRNA expression (Figure 2f–l, Supplementary Fig S2 A,B) in a dose-dependent manner (Figure 2h-l). IDO1 protein expression was induced within 6 hours and sustained for at least 4 days after IFNγ treatment (Figure 2j,k). This data supports the bioinformatic analysis, indicating that IFN-signaling directly induces IDO1 and TRAIL in endothelial cells.

### IDO1 expression in tumor endothelial cells is specifically enhanced by agonistic CD40 mAb therapy and correlates to intratumoral expression of IFNγ

**Figure 3.**
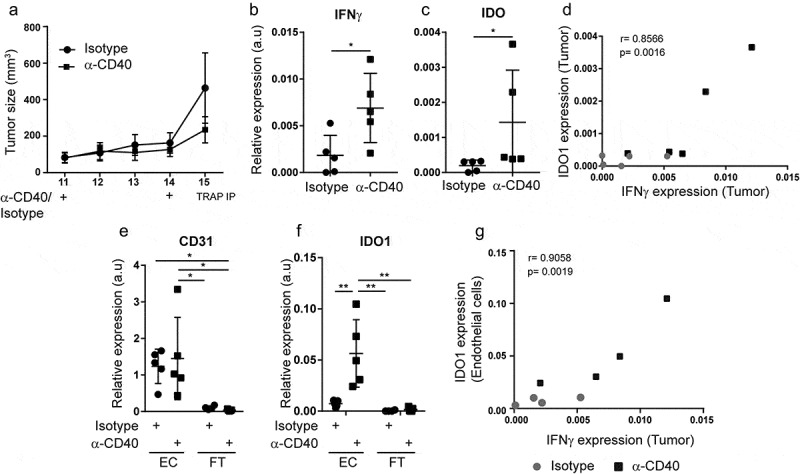
Agonistic CD40 mAb-induced IFNy expression in B16-F10 tumors promotes the expression of IDO1 and TRAIL in tumor endothelial cells: (a) B16-F10 tumor growth curves in mice expressing the fusion ribosomal protein eGFP-L10a under the endothelial specific promoter of VE-cadherin (VEcad-eGFP-L10a TRAP mice). Mice were treated twice with agonistic CD40 mAb (n = 5) or isotype control antibody (n = 5) by peritumoral injections on days 11 and 14. Tumor endothelial-specific mRNA isolation was performed by translating ribosome affinity purification (TRAP-IP). (b-c) qPCR detection of IFNγ (b) and IDO1(c) gene expression in B16-F10 tumors treated with anti-CD40 (n=5) or isotype control (n=5). (d) Pearson correlation (p = 0.0016) of IFNγ and IDO1 expression in B16-F10 tumors treated with anti-CD40 (black boxes) or isotype control (grey dots). (e-f) qPCR detection of CD31 (e) and IDO1 (f) expression in TRAP-purified endothelial cell-specific mRNA (EC) and flow-through (unbound total RNA; FT) samples derived from agonistic CD40 mAb (n = 5) or isotype control (n = 5) treated B16-F10-tumors inoculated in VEcad-eGFP-L10a TRAP mice.(g) Pearson correlation (p = 0.0019) of IFNγ and endothelial-specific IDO1 expression in B16-F10 tumors treated with anti-CD40 (black boxes) or isotype control antibody (grey dots). qPCR graphs depict relative expression of the gene of interest compared to Hypoxanthine-Guanine Phosphoribosyltransferase (HPRT) housekeeping gene expression. Mean, SD, *p < 0.05, **p < 0.01, unpaired Student's t-test, one-way ANOVA with Tukey's multiple comparison test.

IDO1 can be expressed by several cell types within the tumor microenvironment. To determine the relative expression level of IDO1 in endothelial cells as compared to the rest of the tumor tissue, we employed mice expressing a fusion eGFP-L10a ribosomal protein under control of the VE-cadherin promoter (VEcadTRAP mice). VEcadTRAP mice with B16.F10 tumors were treated peritumorally with agonistic CD40 mAb at day 11 and 14, and tumors were collected at day 15 when tumor sizes did not significantly differ between the groups (Figure 3a). Agonistic CD40 mAb treatment substantially increased the expression of IFNγ and IDO1 in tumor tissue, and their levels in individual tumors were positively correlated (Figure 3b–d). The translating-ribosome affinity purification (TRAP) method was used to isolate the endothelial-specific mRNA fraction associated with eGFP-tagged ribosomes and the unbound (flow-through) fraction containing all remaining RNAs from the tumor tissue (including RNA from tumor cells, stroma and immune cells).^32^ Successful isolation of endothelial mRNA was confirmed by qPCR analysis, showing that the endothelial marker CD31 was highly enriched in the eGFP-immunoprecipitated fraction (Figure 3e). IDO1 expression was specifically increased in the tumor endothelial pool following anti-CD40 treatment of B16-F10 tumors (Figure 3f), whereas it was almost undetectable in the unbound fraction. The increase in IDO1 in B16-F10 tumor endothelial cells treated with agonistic CD40 mAb correlated with IFNγ expression in the tumor tissue (Figure 3g). A predominant expression of IDO1 in the tumor endothelial cell fraction was also noted in HCmel12 melanomas, but the level of IDO1 and of intratumoral IFNγ were not significantly increased by anti-CD40 therapy in this model (Supplementary Fig S3A-D). To compare expression of IDO1 in tumor endothelial cells to that of tumor-infiltrating leukocytes, B16-F10 tumor bearing mice were treated with agonistic CD40 mAb or isotype control and CD45^−^CD31^+^ endothelial cells and CD45^+^ leukocytes were isolated using FACS before the start of therapy (day 12), one day after the last treatment (day 16) and four days after the last treatment (day 19). In line with our analysis of the endothelial-specific fraction using the TRAP methodology (Figure 3f), IDO1 mRNA expression was significantly elevated on day 16 in tumor endothelial cells from agonistic CD40 mAb treated mice as compared to expression in tumor endothelial cells before treatment (day 12) and tumor endothelial cells isolated from isotype control treated mice (Supplementary Fig S4A, S4B). The increased expression of IDO1 in tumor endothelial cells in response to agonistic CD40 mAb therapy was sustained in some, but not all, treated mice at day 19. In contrast, agonistic CD40 mAb therapy did not enhance IDO1 expression in CD45^+^ leukocytes, and the relative level of expression was considerably lower in leukocytes than what was observed in tumor endothelial cells after agonistic CD40 mAb therapy (Supplemental Fig S4C). Together, our data indicate that IDO1 is mainly expressed in tumor endothelial cells of B16-F10 and HCmel12 melanomas and that its expression can be further enhanced by agonistic CD40 mAb therapy and the associated IFNγ expression in the tumor microenvironment.

### T-cell secretion of IFNγ enhances IDO1 expression in endothelial cells

**Figure 4.**
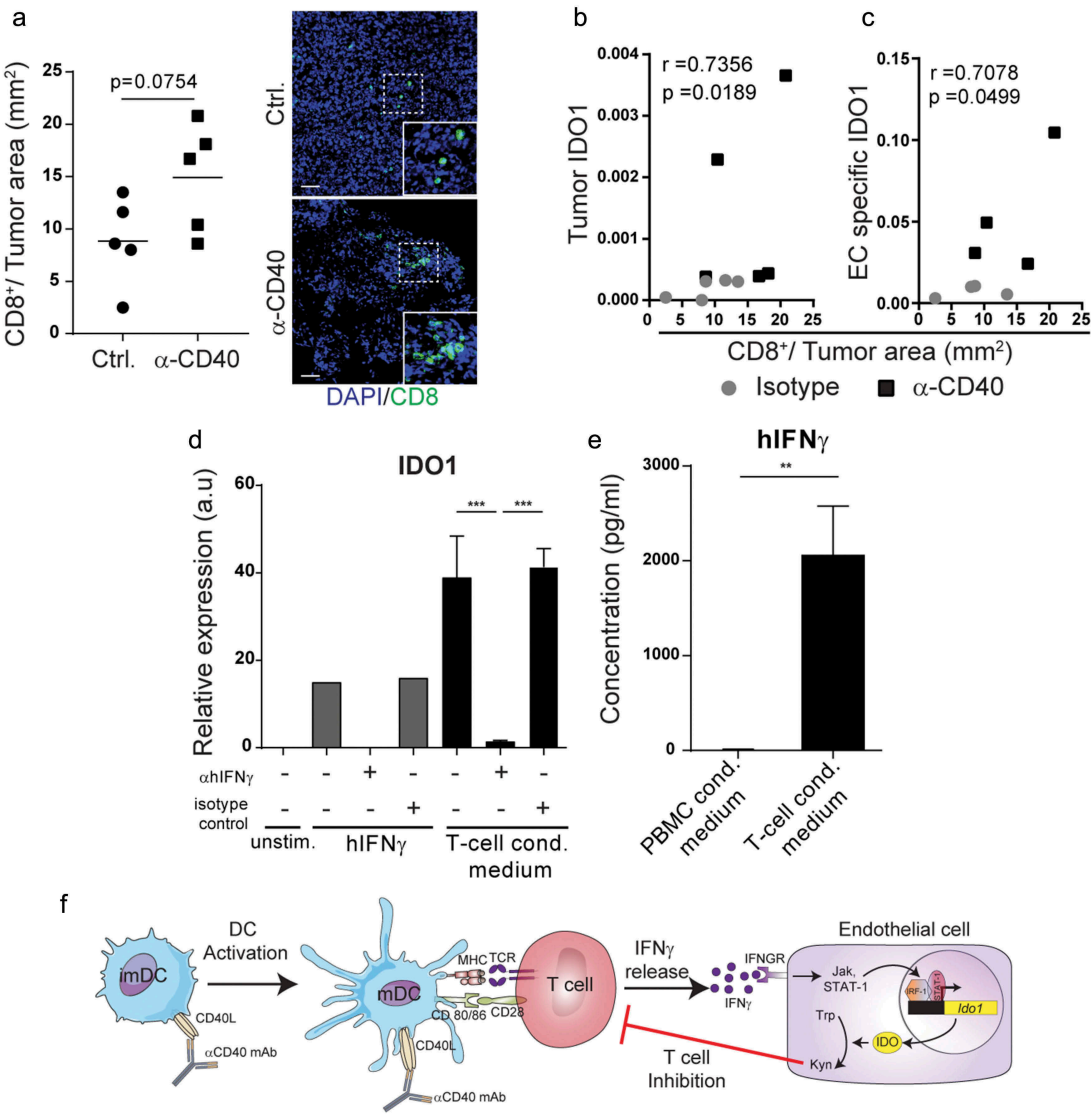
IDO1 expression can be induced by T-cell-derived IFNγ in endothelial cells: (a) CD8^+^ T cell immunofluorescence staining quantification in cryosections from B16-F10 tumors treated with agonistic CD40 mAb or isotype control antibody. Representative immunofluroscence pictures of CD8 staining. Scale bar 50μm. (b) Spearman correlation (p = 0.0189) of tumor IDO1expression and CD8^+^T cells/tumor area in B16-F10 tumors treated with anti-CD40 (black boxes) or isotype control (grey dots). (c) Pearson correlation (p = 0.0499) of endothelial cell-specific IDO1 (derived from the endothelial cell fraction of the TRAP IP) expression and CD8^+^ T cells/tumor area in B16-F10 tumors treated with agonistic CD40 mAb (black boxes) or isotype control antibody (grey dots). Gene expression values show relative expression of IDO1 compared to HPRT housekeeping gene expression (qPCR). (d) IDO1 expression in HDBEC treated for 18 hours with 100ng/ml human recombinant IFNγ or media from 3-day cultured T-cells (3 donors) supplemented with anti-hIFNγ blocking antibody or respective isotype control (both 10μg/ml). Graph shows mean relative expression values compared to HPRT. (e) Detection of IFNγ by ELISA in the T-cell conditioned media (3 healthy donors) that was used to stimulate HDBEC. (f) Schematic illustration of endothelial IDO1-upregulation in response to IFNγ secreted by T-cells.

Since activation of T-cells is associated with enhanced IFNγ production, we investigated if IDO1-expression in tumor endothelial cells in response to agonistic CD40 mAb immunotherapy was related to increased infiltration of activated T-cells. We quantified CD8^+^ T cells by immunofluorescent staining and image analysis and correlated the results to IDO1 expression in the corresponding tumor and in tumor endothelial cells (endothelial fraction of the TRAP immunoprecipitation), and found a positive correlation in both cases (Figure 4a–c). To determine if IFNγ expressed by activated T cells was sufficient to upregulate IDO1 in HDBEC, we isolated T-cells from healthy human donors using CD3-beads. Treatment of HDBEC with T-cell conditioned media was associated with enhanced expression of IDO1, an effect that was abrogated by adding an anti-hIFNγ blocking antibody (Figure 4d). Secretion of IFNγ from the purified CD3^+^ T cells was confirmed by ELISA (Figure 4e). These results are consistent with previous observations demonstrating that the transcription factors STAT1 and IRF1, which are activated in response to IFNγ signaling, bind to the promoter region of IDO1 and regulate gene expression.^33^ Taken together, our results indicate that anti-CD40-therapy induces activation of T-cells in the tumor microenvironment, and that IFNγ secreted by activated T-cells induces up-regulation of IDO1 in tumor endothelial cells (schematically illustrated in Figure 4f).

### Agonistic CD40 mAb in combination with the IDO1 inhibitor epacadostat reduces B16-F10 melanoma growth and enhances T-cell activation

**Figure 5.**
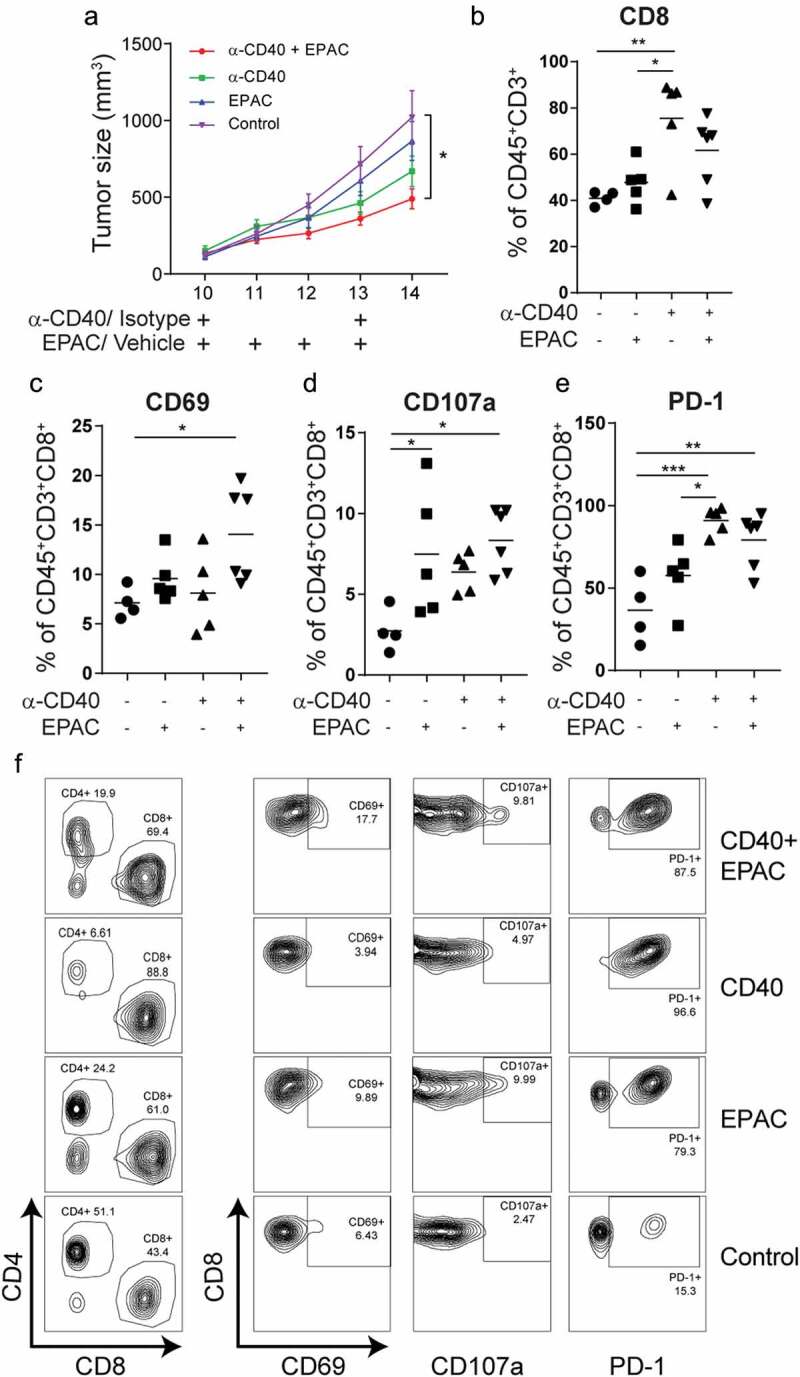
Combining agonistic CD40 mAb treatment with the IDO1 inhibitor epacadostat delays B16-F10 tumor growth and increases survival of tumor-bearing mice: (a) Tumor growth curves in mice bearing B16-F10 tumors treated with agonistic CD40 mAb and epacadostat either as monotherapy or in combination or vehicle as control. Agonistic CD40 mAb or rat isotype control antibody was administered peritumorally on day 10 and day 13 after tumor injection. In parallel,100mg/kg of epacadostat was administered via oral gavage twice daily from day 10 to day 13. Graph shows the combined data from two end-point experiments. Mean, SEM. (b) Percentages of intratumoral CD8^+^ T cells of the total number of CD45^+^CD3^+^measured by FACS (mean, *p < 0.05, **p < 0.01, one-way ANOVA). (c-f) FACS analysis of T-cell activation in tumor-infiltrating T-cells. Values depict percentages of CD69^+^ (c), CD107a^+^(d) and PD-1^+^ (e) of CD45^+^CD3^+^CD8^+^ T-cells from B16-F10 tumors and representative FACS plots (f) (mean, *p < 0.05, **p < 0.01, ***p < 0.01, one-way ANOVA).

**Figure 6.**
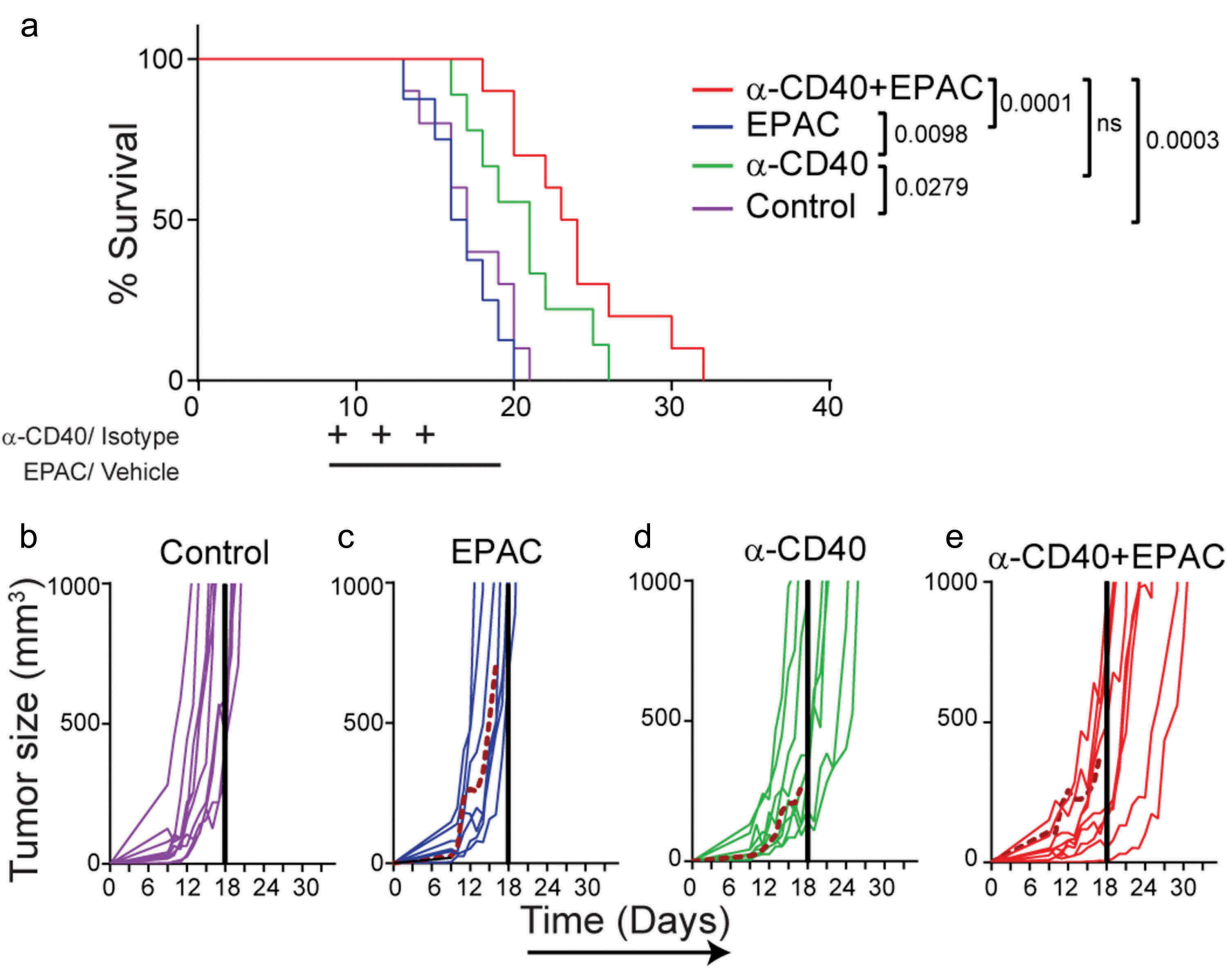
Combined treatment with agonistic CD40 mAb and epacadostat increases survival of B16-F10 tumor bearing mice as compared to control: (a) Kaplan-Meier survival curves of B16-F10 tumor bearing mice treated with agonistic CD40 mAb or isotype control antibody (days 9, 12, 15) and epacadostat or vehicle (from day 9 until 18) (n = 9-10, mean, *p < 0.05, Log-rank test). (b-e) Individual tumor growth curves from the survival experiment in mice bearing B16-F10 tumors of the isotype control treated group (b), or mice treated with either epacadostat (c) or agonistic CD40 mAb (d) or with agonistic CD40 mAb and epacadostat combination (e) therapy. Dashed colored curves represent mice that developed ulcers and the solid colored line indicates the last day of treatment.

IDO1 upregulation in tumor vessels may influence the infiltrating T-cell phenotypes and activation status. Pharmacological inhibition of IDO1 can improve the response to checkpoint antibodies targeting PD-1 or CTLA-4 in B16-F10 melanoma, but it has not yet been tried in combination with CD40-stimulating immunotherapy.^34^ Combining agonistic CD40 mAb therapy with epacadostat significantly reduced B16-F10 tumor growth as compared to control (Figure 5a). The proportion of CD8^+^ T cells within the CD3^+^ T cell population was increased in the anti-CD40 treated tumors as compared to epacadostat or control treated tumors (Figure 5b). Consistent with a role of IDO1 in repressing T-cell activation, the frequency of CD69^+^CD8^+^ activated T cells was increased in tumors treated with the combination therapy compared to control (Figure 5c). In addition, treatment with epacadostat alone or in combination with agonistic CD40 mAb increased the percentage of CD8^+^CD107a^+^ effector T cells whereas PD-1^+^CD8^+^ T cells were increased in groups treated with agonistic CD40 mAb (Figure 5d,e). The representative FACS plots for CD4, CD8, CD69, CD107a and PD-1 expression are shown in Figure 5f. These results indicate that IDO1 inhibition enhances T-cell activation during CD40-stimulating immunotherapy. To determine the effect of combination treatment with epacadostat and agonistic CD40 mAb, mice with palpable tumors were treated with agonistic CD40mAb day 9, day 12 and day 15, and epacadostat was given twice daily from day 9 to day 18. Combining agonistic CD40 mAb with epacadostat significantly prolonged survival of B16-F10 melanoma bearing mice as compared to control and epacadostat alone, while there was no significant difference in survival between mice treated with agonistic CD40 mAb and the combination (Figure 6a). Individual growth curves demonstrate that all mice treated with the combination of agonistic CD40 mAb and epacadostat survived until the last day of treatment (day 18) (Figure 6b–e). These results indicate that combining agonistic CD40 mAb therapy with inhibition of IDO1 is beneficial in mouse melanoma, associated with increased activation of CD8^+^ T-cells.

### Co-treatment with epacadostat reduces the proportion of CD8^+^ T-cells co-expressing inhibitory molecules during CD40-stimulating immunotherapy

**Figure 7.**
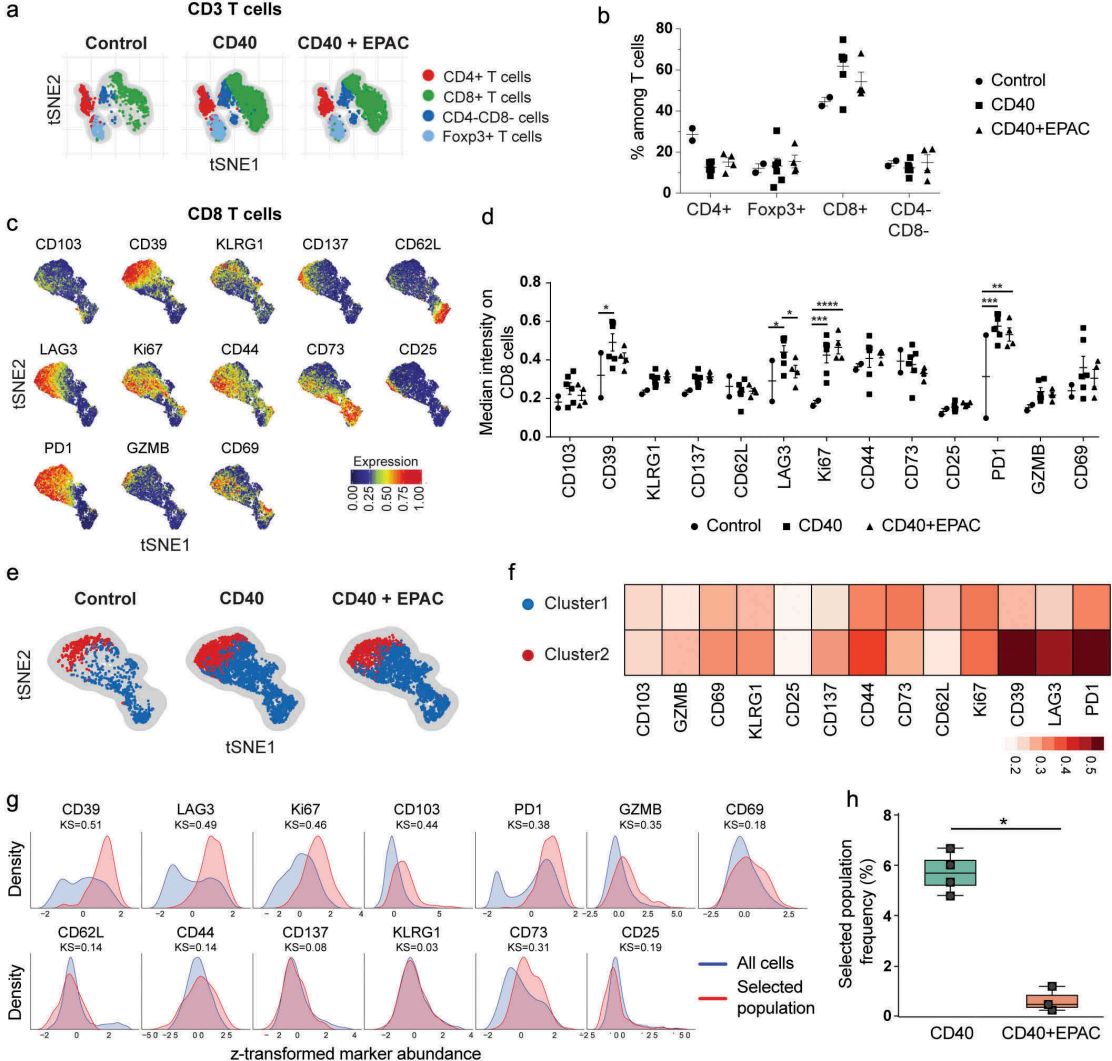
Characterization of tumor infiltrating T cells from B16-F10 tumors treated with agonistic CD40 mAb alone or in combination with epacadostat: B16-F10 tumors were implanted s.c into C57BL/6 mice and treated with control (isotype), agonistic CD40 mAb or agonistic CD40 mAb in combination with Epacadostat. Tumors were harvested at day 15 for flow cytometry analysis . (a-b) t-distributed Stochastic Neighbor Embedding (t-SNE) plot showing sub-populations of TCRβ^+^ T cells (a) and quantification of sub-populations ofTCRβ^+^ T cells from tumors treated by isotype control, agonistic CD40 mAb or agonistic CD40 mAb in combination with epacadostat (b). (c) t-SNE plots showing the median expression of markers on CD8^+^ T cells from tumors of all the three treatments combined. (d) Quantification of median intensity of marker expression on CD8^+^ T cells from tumors treated by isotype control (circles), agonistic CD40 mAb (squares) or agonistic CD40 mAb in combination with epacadostat (triangles).) (mean, *p < 0.05, **p < 0.01, ***p < 0.01, two-way ANOVA and Fischer 's LSD test, each comparison stands alone). (e-f) A pseudocolored t-SNE density map showing the clustering of CD8^+^ T cells from tumors treated with either isotype control, agonistic CD40 mAb or agonistic CD40 mAb in combination with epacadostat (Blue: Cluster PD1^low^LAG3^low^CD39^low^ and Red: Cluster2, PD1^high^LAG3^high^CD39^high^) (e) and heatmap showing median expression of markers in each cluster (f). (g-h) Histograms of the markers (used by CellCnn) showing greatest differential abundance in terms of the Kolmogorov-Smirnov two-sample test between the total CD8^+^ T-cell population and the selected cell subsets (g) and the relative frequency of the selected population in tumors treated with agonistic CD40 mAb or agonistic CD40 mAb in combination with epacadostat (h). (mean, *p < 0.05, Mann-Whitney U-test).

To evaluate how inhibition of IDO1 alters T-cell phenotypes during CD40-stimulating immunotherapy, mice bearing B16-F10 tumors were treated with isotype mAbs, agonistic CD40 mAbs or agonistic CD40 mAbs in combination with epacadostat. Immune cell phenotypes in tumors were determined by high dimensional flow cytometry analysis using a panel of antibodies to characterize cytotoxic and regulatory T-cells. Flow cytometry data were processed using tSNE algorithm and used common T-cell markers to identify the different T-cell subsets present in the tumor (Figure 7a, Supplementary Fig S5A-C) and tumor draining lymph nodes (Supplementary Fig S6A-B). No striking differences were observed in the proportion of helper T-cells (CD4^+^), Foxp3^+^ T-cells and cytotoxic T-cells (CD8^+^) among the different treatment groups in the tumor (Figure 7b). The cytotoxic T-cell (CD8^+^) population was further processed using the tSNE algorithm to identify subpopulations and Figure 7c represents the heatmap intensities of marker expression on CD8^+^ T-cells. Analyzing the median intensity expression of markers on bulk CD8^+^ T-cells reveled that treatment with agonistic CD40 mAb alone or in combination with epacadostat induced T-Cell proliferation (Ki67^+^) and expression of PD-1 as compared to control treated tumors (Figure 7d). Expression of immune-suppressive molecules CD39 and LAG3 on CD8^+^ T-cells decreased when co-treated with epacadostat compared to agonistic CD40 mAb monotherapy. Unsupervised clustering algorithms including FlowSOM and CellCnn were used to identify the population of CD8^+^ T-cells expressing inhibitory molecules. FlowSOM identified a sub-population of PD1^high^LAG3^high^CD39^high^ CD8^+^ T-cells (Cluster-2, in red) that were reduced when agonistic CD40 mAbs were combined with epacadostat (Figure 7e, f). A population of CD8^+^ T-cells with PD1^high^LAG3^high^CD39^high^ signature was also identified using CellCnn. The proportion of these cells were significantly lower in epacadostat co-treated tumors compared to agonistic CD40 mAb monotherapy (Figure 7g,h).

Surprisingly, the proportion of CD4^+^FoxP3^+^ T-cells that co-expressed CD25 was significantly higher in tumors treated by the combination of agonistic CD40 mAb and epacadostat (Supplementary Fig S5D-F). These cells expressed higher levels of immunosuppressive markers, confirming their identity as bona fide regulatory T-cells (Supplementary Fig S5F). No significant differences with respect to the number or phenotype of T-regulatory cells or effector T-cells were detected when comparing leukocytes isolated from lymph nodes of mice treated with anti-CD40 or from the anti-CD40 and epacadostat combination treated group, indicating that the main effect of agonistic CD40 mAb and epacadostat on T-cell activation was observed in the tumor microenvironment (Supplementary Fig S6A-E). Together, these results indicate that inhibition of IDO1, mainly expressed in tumor endothelial cells, reduced expression of immunosuppressive molecules associated with T-cell exhaustion during agonistic CD40 mAb therapy.

## Discussion

Agonistic CD40 mAb therapy have broad immunostimulatory effects on immune cells, but can also impact other cells in the tumor microenvironment. Prior to this study, changes in endothelial gene expression in response to agonistic CD40 mAb therapy had not been investigated. Here, we report that the main response to agonostic CD40 mAb therapy in murine melanoma tumor endothelial cells is not due to engagement of CD40 on endothelial cells, but secondary to IFNγ-secretion by activated cytotoxic T-cells. This leads to simultaneous up-regulation of pro-inflammatory genes, such as CXCL11, and genes associated with an enhanced endothelial barrier to T-cell infiltration and activation, such as IDO1 and TRAIL.^27^ Using the VEcadTRAP mice, we found robust expression of IDO1 in the endothelial fraction in B16-F10 and HCmel12 tumors, while no detectable expression was found in the flow-through. Notably, expression of IDO1 in tumor infiltrating leukocytes was considerably lower than that observed in tumor endothelial cells from agonistic CD40 mAb treated B16-F10 melanomas. This is in line with IDO1 expression in B16-F10 tumors being of stromal origin,^35^ but it does not preclude that specific hematopoietic cell types that are less abundant within the tumor microenvironment may express IDO1. A limitation in this study is that epacadostat is likely to impact all IDO1-expressing cells within the tumor microenvironment, and a specific role of endothelial IDO1 in affecting T-cell activation can only be formally proven by specifically knocking out IDO1 in endothelial cells. Nevertheless, tumor endothelial cells are a main source of IDO1 in B16-F10 and HCmel12 melanomas, and IDO1 expression in tumor endothelial cells is likely to affect the perivascular microenvironment, which all tumor-infiltrating T-cells cross following trans-endothelial migration. Agonistic CD40 mAb treatment increased IDO1 expression in tumor endothelial cells, resulting from increased T-cell infiltration and higher levels of IFNγ in the tumor stroma. This is consistent with depletion of CD8^+^ T-cells or IFNγ-deficiency diminishing IDO1 expression in tumor stroma in murine melanoma.^36^ IFNγ-induced expression of IDO1 in endothelial cells has previously been observed in a model of allotransplantation, where CD40Ig-induced graft acceptance of a complete MHC-mismatched heart was due to Treg induction of IFNγ-induced expression of IDO1 in graft endothelial cells.^37^ The present report is the first to show a specific role of endothelial IDO1 in participating in negative regulation of T-cell activation in response to cancer immunotherapy.

IDO1 has received considerable attention as a possible immunotherapeutic target. Many drugs inhibiting IDO1 been developed and tested in clinical trials. Two drugs currently account for the majority of the trials: indoximod [1-methyl-D-tryptophan (1-DMT)] and epacadostat (INCB024360). Although initial results of IDO1 inhibition in clinical trials were encouraging,^26,38–40^ the recent phase III ECHO 301 trial testing epacadostat in combination with the PD-1 inhibitor pembrolizumab in melanoma did not show an improved outcome as compared to pembrolizumab alone.^41^ In the present study we found that epacadostat improved agonistic CD40 mAb therapy in B16-F10 tumors and enhanced T-cell activation, as evidenced by increased expression of CD69 and CD107a on CD8^+^ effector T-cells. We observed a reduction in CD8^+^ effector T-cells expressing exhaustion markers CD39, PD1 and LAG-3 when agonistic CD40 mAb therapy was combined with epacatostat treatment, indicating that IDO1-inhibition reduced immunosuppression. IDO1 inhibition has been demonstrated to increase conversion of Foxp3^+^ Tregs to Th17-like cells in tumor-draining lymph nodes in mice bearing B16-F10 tumors.^42^ However, when treating mice with epacadostat, we did not detect any significant differences in effector T-cells or T-regulatory cells in the lymph node, and instead the major effects of epacadostat therapy on immune cell phenotypes was observed within the tumor microenvironment. Unexpectedly, we found that CD25^+^FoxP3^+^ Tregs were significantly increased within the tumor microenvironment when agonistic CD40 mAb therapy was combined with epacadostat. Although initially surprising, this observation is consistent with enhanced functionality of effector T-cells, since recruitment of CCR4-expressing Tregs have been shown to occur through secretion of CCL22 by activated CD8^+^ T-cells.^36^ Combining several immunotherapeutic drugs will be necessary to combat the numerous feedback mechanisms that exist to limit an immune response. Nevertheless, our results indicate an advantage of combining agonistic CD40 mAb therapy with IDO1-inhibitors, and support continued development of IDO1-inhibitors or alternative targeting of the Trp-Kyn-AhR pathway to block compensatory mechanisms from other tryptophan catabolizing enzymes.^43^ The present study also indicates that combining IDO1 vaccination with agonistic CD40-therapy might represent an attractive approach to treat cancer. If a T-cell response toward IDO1 could be achieved and supported by agonistic CD40 therapy, this would have the dual benefit of targeting tumor endothelial cells and, in IDO1-expressing tumors, malignant cells. Supporting this concept, T-cell targeting of tumor endothelial cells using CAR T-cells specific for VEGFR2 have resulted in inhibition of tumor growth and prolonged survival in mouse tumor models.^44^

Taken together, our data indicate that tumor endothelial cells harbor immunosuppressive feedback mechanisms that are triggered as a result of T-cell activation and can dampen the response to cancer immunotherapy. It is highly likely that this feedback is not limited to CD40-stimulating immunotherapy, but would be induced by other treatments that induce tumor localized T-cell interferon production such as checkpoint-inhibitor therapy or CAR T cells. It is known that tumor endothelial cells can limit T-cell infiltration by reduced expression of adhesion molecules and chemokines important for capture, firm adhesion and transendothelial migration of leukocytes.^45^ Here we report that increased interferon signaling in the tumor microenvironment leads to endothelia up-regulation of an array of genes that each can dynamically influence immune response and may constitute new targets for therapy, which warrants further investigation.

## Materials and methods

### Cell culture

Primary Human Dermal Blood Endothelial Cells (HDBEC) (PromoCell) and Human Umbilical Vein Endothelial Cells (HUVEC) (3 H Biomedical) were cultured in Endothelial Cell Growth Medium 2 (EMV2) (PromoCell) in gelatin-coated plates. Murine B16-F10 melanoma cells (ATCC CRL-6475, American Type Culture Collection) were cultured in Dulbecco’s Modified Eagle Medium (DMEM) supplemented with GlutaMAX (Thermofisher Scientific) and 10% fetal bovine serum (FBS) (Sigma-Aldrich). HCmel12 murine melanoma cells^46,47^ were generously provided by Prof. T. Tüting (Laboratory of Experimental Dermatology, University of Bonn, Germany) and cultured in RPMI 1640 Medium (Thermofisher Scientific) with 10% FBS. Cell cultures were incubated at 37°C with 5% CO_2_ in a humidified cell incubator. No authentication of cell lines has been performed after purchase. All cell cultures were routinely tested negative for mycoplasma contamination using the MycoAlert Detection Kit (Lonza, Basel, Switzerland).

### Experimental tumor models

B16-F10 melanoma cells or HCmel12 melanoma cells (250.000 cells) were subcutaneously (s.c.) implanted in C57BL/6 male mice (8 weeks old, Taconic Biosciences). Alternatively, for tumor endothelial-specific mRNA extraction by translating ribosome affinity purification (TRAP),^32^ adult transgenic bacTRAP mice carrying the fusion ribosomic protein eGFP-L10a under control of the VE-cadherin promoter were employed (VEcadTRAP mice). The mice were treated with peritumoral injections of 30 µg αCD40 (FGK4.5, BioXcell) or 30 µg rat IgG2a (2A3, BioXcell) isotype antibodies starting when tumors became palpable. In experiments evaluating IDO1-inhibition, 100 mg/kg of Epacadostat (MedChemExpress) dissolved in vehicle (3% N, N-dimethylacetamide, 10% 2-hydroxylpropyl-β-cyclodextrin) was administered via oral gavage twice daily. Mice were sacrificed one day after the last treatment and tumors were analyzed as described below. A detailed description of treatment schedules is available in the Supplemental Methods section.

Tumor growth was monitored with a caliper and tumor volume was calculated using the ellipsoid formula: 4/3 x π x a (radius of length) x b (radius of width) x c (radius of depth). For survival experiment, mice were sacrificed either when tumors reached a volume of 1000 mm^3^ or if mice developed ulcers. The animal experiment was performed according to Uppsala University’s guidelines for animal experimentation (ethical permits C1/14 and C26/15).

### FACS sorting and RNA preparation followed by deep sequencing

The tumors were cut in small pieces and enzymatically digested by a solution containing DMEM (ThermoFisher) + 5 mg/mL Collagenase II (Sigma) + 50 μg/mL DNase I (Sigma) for 40 min at 37°C. After passing through 70 μm cell strainers, the generated single cell suspensions underwent erythrocyte lysis on ice. The samples were stained using the following antibodies in 2 μg/mL in FACS buffer (PBS + 2% BSA + 1 mM EDTA): anti-CD45-APC (30-F11, Biolegend), anti-CD31-PE (MEC 13.3, BD Biosciences). The samples were stained for live/dead staining with DAPI and sorted in a BD FACSAriaIII cytometer (BD Biosciences). The sorted cells (approximately 100.000 CD45^−^CD31^+^ tumor endothelial cells) were collected in PBS and lysed in Qiazol reagent (Qiagen). Total RNA was extracted from tumor endothelial cells isolated from B16-F10 tumors treated with agonistic CD40 mAb or isotype control antibodies using the RNeasy Mini and Microkits (Qiagen). According to the manufacturer´s instructions in the Tru-Seq RNA library preparation kit v2, 10 ng of RNA was converted into cDNA libraries, which were sequenced on the HiSeq 1500 system and demultiplexed using CASAVA v1.8 (Illumina).

### Preprocessing of sequenced data

RNA-seq data were preprocessed and analyzed. All reads were aligned against the murine mm10 reference genome by TopHat2 v2.0.11^48^ using default parameters. The data were imported in Partek Genomics Suite v6.6 (PGS) to deduct gene and transcript information before performing normalization using statistical software R (v3.3.1) and the DESeq2 package (http://dx.doi.org/10.1101/002832). Normalized read counts were floored to a value of at least 1 after correcting for variance introduced by harvest day. Subsequent to flooring, datasets were trimmed by defining a gene as expressed if the maximum value over all group means was higher than 10.

### Identification of differentially expressed genes

A two-way ANalysis Of Variance (ANOVA) using PGS was performed to calculate differentially expressed genes within datasets comparing expression in tumor endothelial cells and macrophages isolated from agonistic CD40 mAb and isotype treated tumors. A manuscript describing the macrophage expression data is under preparation. Genes were defined to be DE when having a fold change (FC) of ≥ 1.5 and an unadjusted *p*-value of ≤ 0.05.

### Gene set and gene ontology enrichment analysis

Gene Set Enrichment Analysis (GSEA) was performed by PGS using 10,000 permutations utilizing the hallmark pathway gene-sets (http://software.broadinstitute.org/gsea/msigdb/index.jsp) to find enriched pathways in anti-CD40 treated tumor endothelial cells compared to isotype controls. Gene Ontology Enrichment Analysis (GOEA) was conducted using the GO biological process terms. Genes upregulated (FC: ≥ 1.5; unadjusted *p*-value of ≤ 0.05) were used as input for the analysis. To visualize the data, BiNGO,^49^ Enrichment Map^50^ and Word Clouding^51^ plug-ins in Cytoscape were employed using default parameters.

### Reverse transcription quantitative PCR (Rt-qPCR)

For total tissue analysis, RNA extraction was performed using 30 cryosections (10 μm) of OCT-embedded tumors (Cat. 45830, Histolab OCT Cryomount), following the protocol of the RNeasy Plus Mini kit (Qiagen). Then, cDNA was prepared using the SuperScript III Reverse Transcriptase kit (Thermofisher Scientific). Quantitative PCR was performed using SYBR Green (Applied Biosystems) in technical duplicates (CFX96 Touch Real-Time PCR Detection System, Biorad). Human (h) or murine (m) hypoxanthineguanine phosphor ribosyltransferase (HPRT) housekeeping gene was used as reference gene depending on the organism analyzed. List of primers used are provided in the Supplemental Data section (Sup. Table S1). All primers were purchased from Thermofisher Scientific. Relative expression (RE) was determined with the formula RE _gene x_ = 2^− (Cq x-Cq gene HPRT)^.

### Tumor endothelial-specific mRNA isolation by translating ribosomal affinity purification

The Translating Ribosomal Affinity Purification (TRAP) protocol was previously used for identification of cell-specific expression.^32^ In the TRAP mice used in this study, the transgene eGFP-L10a is regulated by the endothelial-specific VE-cadherin promoter. Isolation of eGFP-tagged-polysomes was performed by using anti-GFP-bound Dynabeads (Qiagen). After extensive washing, mRNA of endothelial cells from B16-F10 or HCmel12 tumors were obtained by Trizol and further purified by RNeasyMicro kits (Qiagen). Two different RNA pools, 1) the endothelial specific mRNAs of the tumor that were about to be translated and 2) a pool of unbound fraction (flow-through that did not bind to the anti-GFP beads), consisting of the rest of RNAs of the tissue, were collected.

### Cytokines and antibodies for *in vitro* stimulation or endothelial cells

The following cytokines and antibodies were used for *in vitro* stimulation of HDBEC: hIFNγ (Biosite), MegaCD40L (Enzo lifesciences), hIFNγ neutralizing antibody (BD biosciences) and the isotype control for the hIFNγ-neutralizing antibody (BD biosciences). All cytokines and antibodies used in *in vitro* HDBEC stimulations were diluted in endothelial cell starvation medium (basal EMV2 plus 1% FBS). HDBEC were pre-starved for 2 hours in starvation medium prior to stimulation.

### Culture of HDBE cells in T-cell conditioned media

Peripheral Blood Mononuclear Cells (PBMCs) were isolated after Ficoll (GE Healthcare) separation from healthy donor buffy coats. Purified T cells were obtained with anti-human CD3 microbeads (Miltenyi) according to manufacturer’s protocol. CD3^+^ T cells were cultured for 3 days in RPMI supplemented with 10% FBS, 1% PEST, 1% HEPES, 0.5% L-glut, 0.04% b-2-Mercaptoethanol. T-cell conditioned medium (cell culture supernatant) was collected and added to HDBEC monolayers in 24-well plates (70.000 cells/well). IFNγ was performed by adding anti-hIFNγ antibodies and the experiment was controlled using respective isotype antibodies.

### Western blot and ELISA for protein analysis

Cell lysates from HDBEC cultured in gelatin-coated 12-well plates were prepared using a mixture of NuPAGE LDS Sample Buffer and NuPAGE Sample Reducing Agent (Thermofisher Scientific). Samples were loaded on NuPAGE Bis-Tris4%–12% protein gels. NuPAGE MOPS SDS Running Buffer supplemented with 200 μl of NuPAGE Antioxidant was used during electrophoresis, and the gels were transferred using NuPAGE Transfer Buffer. SeeBlue Pl2 USD Pre-Stained Standard (ThermoFisher Scientific) was used as a loading marker. Proteins were blotted onto an Amersham nitrocellulose blotting membrane 0,2 μm-0,45 μm and Amersham ECL prime was used as a detection reagent (GE Healthcare Sciences). Primary antibodies were anti–IDO (D5J4E) Rabbit mAb (Cell Signaling Technology) and Mouse Anti-β-Catenin (Clone 14/Beta-Catenin, BD Biosciences). Horseradish peroxidase-labeled secondary antibodies (GE Healthcare and Sigma) were used. For detection of hIFNγ in isolated T cells from PBMCs from healthy donors, the human IFNγ ELISA development kit (MabTech) was used.

### Flow cytometry analysis

Tumors were cut in small pieces, enzymatically digested with by ± 2.3 Wunsch units/ml Liberase TL (SigmaAldrich) for 20 minutes at 37 °C and passed through 70 μm cell strainers. The generated single cell suspensions were stained with the live/dead marker Zombie Aqua (Biolegend) and blocked for unspecific binding to CD16/32 (TruStain fcX, Biolegend). Single cell suspensions were incubated for 20 minutes with FACS buffer (PBS supplemented with 1% FCS, 0,02% NaN3) with 1:50 dilution for Abs. The antibodies used were purchased from Biolegend: PerCP anti-mouse CD45 (30-F11), Brilliant Violet 421 anti-mouse CD3 (17A2), PE anti-mouse CD4 (RM4-4), APC/Cy7 anti-mouse CD8a (53–6.7), FITC anti-mouse/human CD45R/B220 (RA3-GB2), PE anti-mouse CD69 (H1.2F3), APC/Cy7 anti-mouse CD107a (1D4B) and PE/Cy7 anti-mouse PD-1 (RMP1-30). Samples were washed with FACS-buffer and analyzed in a FACSCanto II cytometer (BD Biosciences). Data analysis was performed with FlowJo software (TreeStar). For high dimensional FACS analysis data was acquired on a FACSymphony using the antibodies described in Sup Table S2. For tSNE and FlowSOM analysis data were compensated, exported into FlowJo software (version 10, TreeStar Inc.). The exported FCS files were normalized using Cyt MATLAB (version 2017b) and uploaded into Rstudio (R software environment, version 3.4.0). tSNE and FlowSOM algorithm mapping live T cells from a pooled sample were performed as described by Brummelman et al (In press, Nat. Protocol). CellCnn was run using default parameters, dividing data into training and validation steps^52^.

### Immunofluorescence staining and image analysis

Cryosections (7–10 μm) from snap frozen tumors were fixed with ice-cold acetone for 15 min and blocked with 3% bovine serum albumin in PBS for 1 h at room temperature. The sections were incubated overnight at 4°C with APC anti-mouse CD8a (Biolegend). After washing with PBS sections were counterstained with Hoechst33342 (SigmaAldrich) and mounted with Fluoromount-G (Southern Biotechnology). Tile-scan images from entire tumor sections were captured using a DMi8 Leica microscope. Cells were counted manually using the Image J software.

## Statistical analysis

Statistical analysis was performed using the GraphPad Prism 7.0 software. Data was assessed for normal distribution using D’Agostino & Pearson normality test. To determine statistically significant differences (*p* value ≤0.05) between groups we used t-student tests and one-way ANOVA (or Kruskal-Wallis test for not normally distributed data) followed by correction for multiple testing (recommended post-hoc test). To determine significant correlations between variables we employed Pearson and Spearman correlation analyses (*p* value ≤ 0.05).

## Data Availability

The mRNA sequencing data has been deposited in NCBI’s Gene Expression Omnibus and are accessible through GEO Series accession number GSE130679 (https://www.ncbi.nlm.nih.gov/geo/query/acc.cgi?acc=GSE13067)
