# Supplementary material for: Tumor endothelial cell up-regulation of IDO1 is an immunosuppressive feed-back mechanism that reduces the response to CD40-stimulating immunotherapy
Source: Oncoimmunology. 2020 Mar 9;9(1):1730538. doi: 10.1080/2162402X.2020.1730538 (PMC7094447; doi:10.1080/2162402X.2020.1730538)
Supplement: Supplemental Material [file koni-09-01-1730538-s001.zip › Supplementary_Methods latest.docx]

**SUPPLEMENTARY DATA**

***SUPPLEMENTARY METHODS***

**Treatment schedules for tumor experiments**

***FACS sorting experiments***

B16.F10 melanoma cells (250.000 cells) were subcutaneously (s.c.) implanted in C57BL/6 male mice (8 weeks old, Taconic Biosciences). The mice were randomly divided into two groups and were treated with 2 peritumoral injections of 30µg αCD40 (FGK4.5, BioXcell) or 30µg rat IgG2a (2A3, BioXcell) isotype antibodies at day 13 and 16 after B16.F10 tumor inoculation. The mice were sacrificed one and four days after the last treatment and tumors were dissected and subjected to FACS sorting of tumor endothelial cells and TILs, followed by RNA-sequencing or qPCR.

***Tumor endothelial-specific mRNA extraction experiments***

For tumor endothelial-specific mRNA extraction by translating ribosome affinity purification (TRAP) (34), adult transgenic bacTRAP mice carrying the fusion ribosomic protein eGFP-L10a under control of the VE-cadherin promoter were produced (VEcadTRAP mice). B16.F10 or HCmel12 melanoma cells (250.000 cells) were s.c. injected into the right flank of adult VEcadTRAP mice. When tumors became palpable (day 11 for B16.F10 and day 17 for HCmel12), mice were treated with either anti-CD40 antibodies or respective isotypes as described above. Mice were sacrificed one day after the last treatment, in B16.F10 melanoma at day15 and in HCmel12 at day 21. The tumors were removed and split in half. Half of the tumor was used for mRNA analysis using the TRAP protocol and the other half was snap frozen in isopentane with dry ice and embedded in OCT (Histolab OCT Cryomount) for immunofluorescence staining and total RNA extraction.

***Experiments combining agonistic CD40 mAbs with Epacadostat treatment***

For end-point studies with agonistic CD40 mAbs in combination with the IDO1 inhibitor Epacadostat (MedChemExpress), 8 weeks old C57BL/6 wild-type male mice (Taconic Biosciences) received 250.000 B16.F10 cells in the right flank s.c. The treatments started at day10 after tumor injection, when the tumors were palpable. 30 μg agonistic rat-anti-mouse CD40 antibody (FGK4.5, Bio X Cell) or rat control isotype IgG2a was administered in phosphate-buffered saline (PBS) peritumorally on day 10 and day 13 after tumor injection. In parallel, 100 mg/kg of Epacadostat dissolved in vehicle (3% N, N-dimethylacetamide, 10% 2-hydroxylpropyl-β-cyclodextrin) was administered via oral gavage twice daily from day 10 to day 13. Mice were sacrificed one day after the last treatment (day 14) and the tumors were surgically removed and split in half. One half of the tumor was used for FACS analysis and the other half was snap frozen in isopentane with dry ice for further analyses.

For the survival experiment, 8 weeks old C57BL/6 wild-type male mice (Taconic Biosciences) were s.c. injected with B16.F10 cells as described above. On day 9, when tumors were palpable, the mice were treated with local anti-CD40 antibodies or the isotype controls as described above. The treatment was repeated on day12 and day14. In parallel, 100mg/kg of Epacadostat or vehicle (3% *N*, *N*-dimethylacetamide, 10% 2-hydroxylpropyl-β-cyclodextrin from Sigma) were administered via oral gavage twice a day continuously from day 9 until day 18. Mice were sacrificed either when tumors reached a volume of 1000 mm^3^ or if mice developed ulcers.

***MATERIALS AND METHODS SUPPLEMENTARY FIGURES***

**Cell culture**

The immortalized murine brain capillary-derived bEND.3 and pancreatic islet MS1 endothelial cell lines were cultured in DMEM (Dulbecco's Modified Eagle Medium, Gibco) supplemented with 10% FCS (Gibco). The murine immature D1 cells were cultured in non-tissue culture-treated dishes in IMDM (Iscove's Modified Dulbecco's Medium, Gibco) with 10% FBS, 1% βME and 20ng/ml GM-CSF.

Human monocyte-derived DCs (moDCs) were obtained by isolating CD14^+^ monocytes from healthy donor buffy coats and culturing them in GM-CSF and IL4 (both at 100ng/ml) for 6-7 days. Differentiation of the cells was checked by FACS stain for CD14^+^ and CD1a^+^ on samples pre- and post- CD14^+^ cell isolation as well as after 6-7 days of culture.

***In vitro* cell stimulations**

HDBEC, HUVEC and moDCs were stimulated for 24 hours with hTNFα (PeproTech, 20ng/ml) and human MegaCD40L (1μg/ml, Enzo lifesciences) before FACS staining. D1 dendritic cells, bEND.3 and MS1 and cell lines were stimulated for 24 hours with mTNFα (PeproTech, 20ng/ml), mouse MegaCD40L (1μg/ml, Enzo lifesciences), rat anti-mouse CD40 antibody (FGK4.5) or respective IgG2a isotype (10μg/ml, both from BioXcell) before FACS staining.

**FACS staining**

For FACS staining, single cell suspensions were incubated for 20 minutes with FACS buffer (PBS supplemented with 1% FCS, 0,02% NaN3) with 1:50 dilution for Abs. The antibodies used were: anti-human CD40 (mouse IgG1κ, 5C3, Biolegend), mouse IgG1κ isotype control (Biolegend), anti-human ICAM-1 (HA58, Biolegend), anti-human VCAM-1 (BBA22, R&D), anti-human E-selectin (HA3-1f, Biolegend), anti-human CD86 (IT22, Biolegend), anti-human HLA-DR (L243, BD Biosciences), anti-mouse CD40 (rat IgG2a, 3/23, Biolegend), rat IgG2a isotype control (Biolegend), κanti-mouse ICAM-1 (YN1/1.7.4, Biolegend), VCAM-1 (MVCAM.A, Biolegend) and anti-mouse CD86 (GL-1, Biolegend). Samples were washed with FACS-buffer and analyzed in a FACSCanto II cytometer (BD Biosciences). Data analysis was performed with FlowJo software (TreeStar).
