# Supplementary material for: Tumor endothelial cell up-regulation of IDO1 is an immunosuppressive feed-back mechanism that reduces the response to CD40-stimulating immunotherapy
Source: Oncoimmunology. 2020 Mar 9;9(1):1730538. doi: 10.1080/2162402X.2020.1730538 (PMC7094447; doi:10.1080/2162402X.2020.1730538)
Supplement: Supplemental Material [file koni-09-01-1730538-s001.zip › Table_S1_S2_GeorganakiEtAl.docx]

**Table S1.** Primers for qPCR analysis (h=human, m=mouse)

| **Gene** | **Forward Primer (5'-3')** | **Reverse Primer (5'-3')** |
| --- | --- | --- |
| hHPRT | CTTTGCTGACCTGCTGGATT | TCCCCTGTTGACTGGTCATT |
| hIDO1 | TCTCATTTCGTGATGGAGACTGC | GTGTCCCGTTCTTGCATTTGC |
| mHPRT | CAAACTTTGCTTTCCCTGGT | TCGAGAGGTCCTTTTCACC |
| mIDO1 | TGGCGTATGTGTGGAACCG | CTCGCAGTAGGGAACAGCAA |
| mTRAIL | ATGGTGATTTCGATAGTGCTTCC | GCAAGCAGGGTCTGTTCAAGA |
| mCD31 | TACTGCAGGCATCGGCAAA | GCATTTCGCACACCTGGAT |
| mCD45 | ATATCGCGGTGTAAAACTCGTC | TTCCACAAGGCGTTTCTGGAA |
| mCCL19 | TGGTTCTCTGGACCTT | CCCTTAGTGTGGTGAAC |
| mGBP4 | GGAGAAGCTAACGAAGGAACAA | TTCCACAAGGGAATCACCATTTT |
| mGBP10 | GGTGATTCCTAGGAGAGAG | TGTCCAGAATCTCTATGG |
| mIFNγ | CACGGCACATCATTGAAAG | TTCCACATCTATGCCACTTGA |

**Table S2.** Antibodies used for high dimensional FACS analysis

| **Antigen** | **Clone** | **Fluorochrome** | **Manufacturer** |
| --- | --- | --- | --- |
| CD25 | PC61 | BV 650 | BioLegendTM |
| CD11b | M1/70 | BUV 661 | BD PharmingenTM |
| CD39 | 24DMS1 | PerCP-eFlour710 | Thermo Fisher Scientific |
| CD4 | GK1.5 | BUV 496 | BD PharmingenTM |
| CD44 | IM7 | BV 570 | BioLegendTM |
| CD45 | 30-F11 | BUV 563 | BD PharmingenTM |
| PD-1 | 29F.1A12 | BV 785 | BioLegendTM |
| CD8 | 53-6.7 | BUV 805 | BD PharmingenTM |
| CTLA4 | UC10-4F10-11 | APC-AR700 | BD PharmingenTM |
| FOXP3 | FJK-16s | PE-eFlour610 | Thermo Fisher Scientific |
| Granzyme B | GB11 | PE | BD PharmingenTM |
| CD103 | 2E7 | FITC | BioLegendTM |
| CD62L | MEL-14 | BUV 737 | BD PharmingenTM |
| KI-67 | B56 | BV 480 | BD PharmingenTM |
| KLRG1 | 2F1/KLRG1 | APC-C7 | BioLegendTM |
| LAG3 | C9B7W | BV 421 | BioLegendTM |
| CD137 | 17B5 | Biotin | BioLegendTM |
| CD73 | TY/11.8 | BV 605 | BioLegendTM |
| Streptavidin |  | BUV395 | BD PharmingenTM |
| CD69 | H1.2F3 | PE-Cy7 | BD PharmingenTM |
| TCRb | H57-597 | PE-Cy5 | BioLegendTM |
| TIM3 | RMT3-23 | APC | BioLegendTM |
| NK1.1 | PK136 | BV 711 | BioLegendTM |
|  |  |  |  |
| LIVE/DEAD Fixable Aqua | |  |  |
