# Supplementary material for: Tumor endothelial cell up-regulation of IDO1 is an immunosuppressive feed-back mechanism that reduces the response to CD40-stimulating immunotherapy
Source: Oncoimmunology. 2020 Mar 9;9(1):1730538. doi: 10.1080/2162402X.2020.1730538 (PMC7094447; doi:10.1080/2162402X.2020.1730538)
Supplement: Supplemental Material [file koni-09-01-1730538-s001.zip › Table_S3_Word_File latest.docx]

| Table S3: DE genes in B16.F10-derived TECs (anti-CD40 vs isotype) | | | | | | | |
| --- | --- | --- | --- | --- | --- | --- | --- |
| Gene Symbol | Mean(TECs_anti-CD40) | Std(TECs_ anti-CD40) | Mean(TECs_isotype) | Std(TECs_isotype) | unadj p-value | adj p-value | FoldChange (anti-CD40 vs. isotype) |
| Ubd | 512.424 | 242.761 | 21.8884 | 24.4688 | 0.000110688 | 0.114351 | 23.4108 |
| Gbp10 | 258.952 | 66.1117 | 22.8559 | 10.743 | 4.68E-07 | 0.00655465 | 11.3298 |
| Ido1 | 21.0812 | 6.59064 | 2.49549 | 1.74255 | 6.83E-06 | 0.0191506 | 8.44772 |
| C1s2 | 10.2154 | 1.88186 | 1.39829 | 0.796589 | 0.00838586 | 1 | 7.30564 |
| Ccl19 | 29.536 | 9.054 | 4.07535 | 2.1444 | 2.19E-05 | 0.0323322 | 7.24748 |
| Serpina3f | 278.548 | 92.2077 | 41.9264 | 26.5316 | 7.16E-05 | 0.0835854 | 6.64373 |
| Gm4841 | 69.7893 | 41.3924 | 13.7317 | 10.5434 | 0.00520409 | 1 | 5.08236 |
| Gbp11 | 22.6531 | 12.8899 | 4.56951 | 7.13903 | 0.00488698 | 1 | 4.95745 |
| 2010005H15Rik | 21.5114 | 6.05134 | 4.45042 | 2.98197 | 0.00397109 | 1 | 4.83356 |
| Gbp5 | 824.75 | 234.242 | 179.512 | 129.589 | 0.000135708 | 0.126823 | 4.5944 |
| Cxcl11 | 16.2156 | 9.99911 | 3.53132 | 3.29239 | 0.0401319 | 1 | 4.59194 |
| Gpr88 | 11.512 | 5.88964 | 2.63073 | 1.89462 | 0.00858667 | 1 | 4.37596 |
| Tgtp1 | 1469.12 | 400.022 | 348.415 | 201.776 | 1.85E-05 | 0.0323322 | 4.21656 |
| Cxcl9 | 4073.1 | 1677.91 | 999.675 | 694.912 | 0.00123611 | 0.541495 | 4.07443 |
| Gbp6 | 1564.75 | 357.002 | 397.068 | 243.504 | 1.68E-05 | 0.0323322 | 3.94076 |
| H2-Q9 | 698.211 | 187.13 | 179.642 | 121.708 | 0.00520961 | 1 | 3.88669 |
| Dgkg | 7.86075 | 1.99899 | 2.06749 | 1.20532 | 0.0400538 | 1 | 3.80207 |
| Gbp2 | 2739.25 | 788.428 | 737.274 | 467.117 | 0.00104944 | 0.501225 | 3.71537 |
| Igtp | 1284.21 | 357.716 | 353.71 | 182.877 | 0.00514115 | 1 | 3.63069 |
| Apol6 | 94.0123 | 44.0089 | 25.9598 | 16.6939 | 0.0017093 | 0.630551 | 3.62145 |
| H2-Q7 | 915.658 | 388.022 | 253.054 | 130.861 | 0.0361881 | 1 | 3.61842 |
| Gm5796 | 20.5736 | 10.1818 | 5.86064 | 5.73723 | 0.00391839 | 1 | 3.51047 |
| H2-Q6 | 406.087 | 183.612 | 118.16 | 81.6839 | 0.00483307 | 1 | 3.43675 |
| Tgtp2 | 2020.83 | 486.297 | 588.95 | 264.756 | 0.000186942 | 0.137924 | 3.43125 |
| Gbp4 | 2546.43 | 470.224 | 743.155 | 292.636 | 2.23E-06 | 0.0104184 | 3.42651 |
| H2-Q5 | 250.516 | 76.6052 | 74.0849 | 24.4977 | 0.0101248 | 1 | 3.38147 |
| Iigp1 | 3881.4 | 1078.15 | 1207.18 | 610.127 | 0.00017445 | 0.135858 | 3.21525 |
| 2210039B01Rik | 44.2599 | 11.0475 | 14.3254 | 8.21242 | 0.0047428 | 1 | 3.08961 |
| Muc2 | 24.0886 | 17.9046 | 8.03102 | 6.84301 | 0.0467626 | 1 | 2.99945 |
| Gm12185 | 63.7624 | 23.9621 | 21.4103 | 10.4823 | 0.00655135 | 1 | 2.97812 |
| Nlrc5 | 787.752 | 93.3491 | 271.062 | 142.33 | 0.000166647 | 0.135858 | 2.90617 |
| Zfp185 | 11.7803 | 1.85201 | 4.27013 | 3.0259 | 6.80E-05 | 0.0835854 | 2.75877 |
| Apol10b | 213.944 | 29.5333 | 79.4561 | 30.2165 | 1.42E-06 | 0.0099764 | 2.6926 |
| Hhatl | 10.8702 | 5.77641 | 4.06363 | 2.17101 | 0.0227976 | 1 | 2.67501 |
| Saa3 | 1462.03 | 626.847 | 550.8 | 259.354 | 0.036051 | 1 | 2.65437 |
| Gbp3 | 985.816 | 110.275 | 382.591 | 180.312 | 2.31E-05 | 0.0323322 | 2.57668 |
| Pnck | 18.7665 | 6.64058 | 7.28656 | 4.05634 | 0.0100846 | 1 | 2.57549 |
| Irgm2 | 1066.54 | 164.014 | 416.113 | 127.393 | 0.000587811 | 0.34333 | 2.56311 |
| H2-Q4 | 2210.55 | 490.787 | 862.937 | 353.794 | 0.00208527 | 0.749522 | 2.56166 |
| Tnfsf10 | 682.36 | 113.064 | 267.53 | 84.5571 | 3.33E-06 | 0.0116855 | 2.55059 |
| C3 | 7536.1 | 2435.81 | 2958.29 | 730.355 | 0.00529184 | 1 | 2.54745 |
| Gm4951 | 210.178 | 37.6407 | 82.6366 | 48.2893 | 0.00249763 | 0.853944 | 2.5434 |
| 1500015O10Rik | 14.0696 | 1.97816 | 5.70442 | 3.14004 | 0.017801 | 1 | 2.46643 |
| Gm18853 | 201.736 | 16.8091 | 85.2928 | 28.6722 | 2.29E-05 | 0.0323322 | 2.36522 |
| 3300005D01Rik | 25.4555 | 9.12473 | 10.8264 | 5.47308 | 0.00571726 | 1 | 2.35125 |
| Zc2hc1c | 12.7989 | 3.88128 | 5.47244 | 3.58719 | 0.0446881 | 1 | 2.33879 |
| Wnt16 | 16.3853 | 6.85627 | 7.06174 | 7.21208 | 0.0230248 | 1 | 2.32029 |
| Alx4 | 18.1817 | 4.41297 | 7.98704 | 5.0669 | 0.00109899 | 0.501225 | 2.2764 |
| Gbp7 | 1458.67 | 193.054 | 641.372 | 237.968 | 0.000251909 | 0.176563 | 2.27429 |
| Gm15091 | 20.6342 | 3.32218 | 9.1475 | 11.0319 | 0.0182769 | 1 | 2.25572 |
| Gm17757 | 213.462 | 34.4461 | 94.8736 | 31.8308 | 0.000153922 | 0.134855 | 2.24996 |
| Fam169a | 12.8966 | 6.04919 | 5.82314 | 2.22135 | 0.0379274 | 1 | 2.21472 |
| Stk32c | 44.8264 | 15.9838 | 20.4257 | 12.5359 | 0.00622935 | 1 | 2.1946 |
| Crabp2 | 24.9508 | 6.57779 | 11.3779 | 8.26902 | 0.00928376 | 1 | 2.19292 |
| H2-T10 | 103.431 | 13.4551 | 47.3874 | 19.9252 | 0.037003 | 1 | 2.18268 |
| Vwa7 | 16.4617 | 3.94164 | 7.72676 | 3.45501 | 0.0202817 | 1 | 2.13049 |
| Irgm1 | 1713.63 | 404.796 | 812.342 | 285.384 | 0.0184706 | 1 | 2.10949 |
| Tnfrsf8 | 21.536 | 5.58997 | 10.2183 | 9.25836 | 0.0158748 | 1 | 2.1076 |
| Sema6c | 28.132 | 9.12311 | 13.6204 | 9.17711 | 0.0241171 | 1 | 2.06543 |
| Serpina3i | 24.5177 | 3.0646 | 11.9484 | 7.44584 | 0.00356388 | 0.96074 | 2.05196 |
| Apol9a | 104.592 | 20.5265 | 51.1641 | 24.6702 | 0.0029764 | 0.906141 | 2.04424 |
| Crhbp | 16.6277 | 7.96334 | 8.24853 | 3.42047 | 0.0181879 | 1 | 2.01583 |
| Hoxd3 | 29.2811 | 3.44213 | 14.8544 | 11.0338 | 0.00458276 | 1 | 1.97121 |
| Vmn2r29 | 16.7274 | 4.24389 | 8.57961 | 2.83285 | 0.0325363 | 1 | 1.94967 |
| Cml3 | 19.1223 | 6.02239 | 10.0445 | 8.73554 | 0.0375646 | 1 | 1.90375 |
| Tc2n | 46.6727 | 15.1491 | 24.7813 | 13.0084 | 0.00955136 | 1 | 1.88338 |
| Lrrtm2 | 21.5928 | 7.35808 | 11.6025 | 5.49197 | 0.0116151 | 1 | 1.86104 |
| Rhbdl3 | 22.8324 | 7.22715 | 12.36 | 8.07151 | 0.0196458 | 1 | 1.84728 |
| Gvin1 | 3553.72 | 542.495 | 1928.66 | 489.58 | 0.00106122 | 0.501225 | 1.84259 |
| Gm4070 | 3593.99 | 535.086 | 1961.5 | 498.211 | 0.00110843 | 0.501225 | 1.83226 |
| Stat1 | 2981 | 493.905 | 1634.76 | 600.997 | 0.0118136 | 1 | 1.82352 |
| Greb1l | 13.6487 | 7.13246 | 7.55834 | 3.06607 | 0.0466482 | 1 | 1.80578 |
| Rasef | 13.5318 | 5.1381 | 7.55699 | 5.14612 | 0.0386198 | 1 | 1.79063 |
| Casq1 | 15.8994 | 4.30534 | 8.88661 | 6.68769 | 0.0373811 | 1 | 1.78914 |
| Gata6 | 28.7287 | 9.01436 | 16.25 | 7.2795 | 0.0116262 | 1 | 1.76792 |
| C1ra | 1594.88 | 313.042 | 902.793 | 395.712 | 0.00275642 | 0.878171 | 1.7666 |
| Draxin | 32.2076 | 9.23654 | 18.2418 | 11.3251 | 0.0282589 | 1 | 1.76559 |
| Tmtc1 | 124.078 | 30.0718 | 70.6488 | 20.9245 | 0.00152685 | 0.578472 | 1.75626 |
| C130083M11Rik | 14.2379 | 3.70986 | 8.11527 | 4.41677 | 0.0145807 | 1 | 1.75445 |
| Clip4 | 45.1957 | 15.6002 | 25.7707 | 13.216 | 0.0418169 | 1 | 1.75376 |
| Chga | 152.612 | 42.0783 | 87.547 | 29.7347 | 0.00493861 | 1 | 1.7432 |
| Clic5 | 316.778 | 96.102 | 183.201 | 35.352 | 0.0039596 | 1 | 1.72913 |
| Car8 | 38.4757 | 10.9143 | 22.2723 | 6.78036 | 0.00460525 | 1 | 1.72752 |
| S1pr4 | 41.2087 | 8.12073 | 23.8905 | 5.56028 | 0.0304909 | 1 | 1.7249 |
| Tnfsf18 | 370.871 | 118.455 | 215.245 | 69.6623 | 0.00832153 | 1 | 1.72302 |
| 4933417G07Rik | 5.44436 | 1.05207 | 3.17126 | 1.79338 | 0.0313246 | 1 | 1.71678 |
| Lbp | 514.314 | 90.9276 | 301.026 | 123.958 | 0.00303814 | 0.906141 | 1.70854 |
| Col7a1 | 60.2241 | 15.904 | 35.7397 | 15.4211 | 0.011142 | 1 | 1.68508 |
| Eps8l2 | 30.2871 | 7.17241 | 18.0874 | 10.4185 | 0.0188309 | 1 | 1.67449 |
| Sept1 | 60.1155 | 15.5325 | 36.047 | 14.7552 | 0.0127881 | 1 | 1.6677 |
| Noxo1 | 36.2441 | 7.9623 | 21.8003 | 8.23086 | 0.0263677 | 1 | 1.66255 |
| Glt1d1 | 25.5247 | 8.56605 | 15.3558 | 6.88207 | 0.0410984 | 1 | 1.66221 |
| Smad6 | 91.2273 | 19.2402 | 54.8978 | 11.0212 | 0.00324226 | 0.927552 | 1.66177 |
| Rasgrf2 | 80.4159 | 34.1303 | 48.8699 | 9.6513 | 0.040711 | 1 | 1.64551 |
| A530050N04Rik | 12.9529 | 4.50322 | 7.87644 | 3.87226 | 0.0443632 | 1 | 1.64451 |
| Lrch2 | 55.9494 | 18.533 | 34.1619 | 10.1826 | 0.0143054 | 1 | 1.63778 |
| Rorb | 10.4568 | 1.45345 | 6.38876 | 3.16505 | 0.0124326 | 1 | 1.63675 |
| Gbp9 | 1138.4 | 192.804 | 697.528 | 235.395 | 0.0098608 | 1 | 1.63205 |
| Nr1h4 | 25.9619 | 2.11051 | 15.9463 | 5.90588 | 0.00244454 | 0.853944 | 1.62808 |
| C8g | 13.5371 | 4.71851 | 8.34924 | 1.19953 | 0.0492367 | 1 | 1.62136 |
| Fam26e | 53.9982 | 13.2978 | 33.3584 | 7.51754 | 0.00640608 | 1 | 1.61873 |
| Slc12a5 | 41.9243 | 10.4917 | 26.0337 | 6.48599 | 0.00710408 | 1 | 1.61039 |
| Adck3 | 226.129 | 80.2616 | 141.232 | 32.735 | 0.0334076 | 1 | 1.60112 |
| Spire2 | 30.5092 | 8.05711 | 19.1217 | 6.34981 | 0.00928678 | 1 | 1.59553 |
| Serpinb9 | 624.285 | 70.6686 | 393.353 | 141.994 | 0.00286989 | 0.894004 | 1.58709 |
| E130310I04Rik | 11.7071 | 3.3887 | 7.37836 | 1.29342 | 0.0335121 | 1 | 1.58668 |
| Ltbp1 | 240.023 | 72.038 | 151.323 | 43.1833 | 0.012232 | 1 | 1.58617 |
| Serpinb6b | 90.0321 | 14.1181 | 56.974 | 21.7109 | 0.00718491 | 1 | 1.58023 |
| Mustn1 | 102.746 | 29.6482 | 66.036 | 23.8149 | 0.0346143 | 1 | 1.5559 |
| Tspan8 | 33.1902 | 8.77453 | 21.5297 | 6.87051 | 0.023577 | 1 | 1.5416 |
| Gm829 | 20.8406 | 4.6895 | 13.5311 | 3.92375 | 0.0150765 | 1 | 1.5402 |
| Rgs9 | 188.282 | 49.7432 | 122.309 | 41.4428 | 0.0145278 | 1 | 1.53939 |
| Adamts15 | 243.171 | 72.2437 | 158.486 | 63.9983 | 0.0308393 | 1 | 1.53433 |
| Parp12 | 807.791 | 60.3265 | 528.916 | 126.533 | 0.0441494 | 1 | 1.52726 |
| Acss2 | 101.387 | 18.7618 | 66.4524 | 21.5583 | 0.0434317 | 1 | 1.5257 |
| Tmeff2 | 63.6555 | 23.3265 | 41.7323 | 12.5522 | 0.0447879 | 1 | 1.52533 |
| C1s1 | 2620.05 | 424.704 | 1732.77 | 792.42 | 0.0184023 | 1 | 1.51206 |
| Cttnbp2 | 94.7229 | 6.95852 | 62.6705 | 16.8373 | 0.000444902 | 0.283483 | 1.51144 |
| Ifi44 | 449.837 | 60.8458 | 298.395 | 106.282 | 0.0183468 | 1 | 1.50752 |
| Oacyl | 34.5964 | 7.39201 | 52.8643 | 19.1825 | 0.0273153 | 1 | -1.52803 |
| Adamts17 | 22.0201 | 7.34376 | 34.5357 | 13.2583 | 0.0379578 | 1 | -1.56837 |
| Pcsk9 | 42.6821 | 11.8589 | 67.0186 | 15.7949 | 0.00955923 | 1 | -1.57018 |
| Ttll11 | 11.7675 | 2.28717 | 18.5354 | 3.73397 | 0.0302853 | 1 | -1.57513 |
| Efhc1 | 29.3158 | 4.48904 | 46.7016 | 11.648 | 0.00995114 | 1 | -1.59305 |
| Sft2d3 | 62.7515 | 10.0109 | 100.043 | 27.9467 | 0.0213818 | 1 | -1.59427 |
| Zfp365 | 81.593 | 14.012 | 131.905 | 54.5056 | 0.0354123 | 1 | -1.61662 |
| Pcdhgb8 | 17.1537 | 10.0487 | 28.1439 | 7.52722 | 0.0321005 | 1 | -1.64069 |
| Nlrp10 | 20.6511 | 8.53855 | 33.9634 | 8.12195 | 0.017757 | 1 | -1.64463 |
| Dlk1 | 146.249 | 70.9555 | 241.637 | 45.0936 | 0.00761254 | 1 | -1.65223 |
| C530008M17Rik | 46.921 | 10.4337 | 77.8807 | 25.596 | 0.00950002 | 1 | -1.65983 |
| Foxp2 | 28.7962 | 5.69817 | 47.9524 | 20.6176 | 0.0297895 | 1 | -1.66523 |
| 2810468N07Rik | 7.01003 | 1.28886 | 11.8034 | 4.43634 | 0.0126116 | 1 | -1.68378 |
| Ccdc171 | 14.539 | 7.12308 | 24.531 | 5.42049 | 0.0459011 | 1 | -1.68725 |
| Rab39b | 21.6271 | 9.1453 | 36.8613 | 12.0794 | 0.0397936 | 1 | -1.7044 |
| Zfp345 | 13.8967 | 3.99502 | 23.7415 | 8.52603 | 0.0218045 | 1 | -1.70842 |
| Rad54b | 97.8252 | 26.002 | 169.818 | 76.3691 | 0.0443864 | 1 | -1.73593 |
| E330033B04Rik | 18.2302 | 4.11366 | 31.7546 | 6.34573 | 0.0426019 | 1 | -1.74186 |
| Gm2115 | 6.60779 | 1.98064 | 11.5598 | 5.25202 | 0.0308108 | 1 | -1.74943 |
| Mfrp | 6.80205 | 3.53178 | 11.9265 | 5.10442 | 0.0441042 | 1 | -1.75337 |
| Chad | 7.06208 | 3.25512 | 12.3859 | 2.99011 | 0.00680808 | 1 | -1.75386 |
| Cacng1 | 16.1197 | 5.47764 | 28.2948 | 2.65598 | 0.000114204 | 0.114351 | -1.75529 |
| Ccdc138 | 24.8834 | 4.81344 | 43.7075 | 12.5983 | 0.00711953 | 1 | -1.75649 |
| Rph3al | 20.2946 | 6.506 | 35.7108 | 10.9046 | 0.0061026 | 1 | -1.75962 |
| Pabpc1l | 12.2977 | 2.26343 | 21.7867 | 6.6959 | 0.0256223 | 1 | -1.7716 |
| Magi2 | 18.9664 | 6.76267 | 33.8436 | 10.2152 | 0.0368918 | 1 | -1.7844 |
| Svopl | 7.42695 | 3.32495 | 13.2906 | 5.9169 | 0.0326083 | 1 | -1.78951 |
| Mum1l1 | 18.1118 | 5.5833 | 32.4572 | 7.75077 | 0.00141285 | 0.565865 | -1.79205 |
| C030037D09Rik | 7.69495 | 2.17737 | 13.9867 | 4.71805 | 0.0299031 | 1 | -1.81764 |
| Exd1 | 7.45255 | 2.73078 | 13.5774 | 3.8897 | 0.0047966 | 1 | -1.82185 |
| Kazald1 | 9.7823 | 2.44762 | 17.8759 | 9.11997 | 0.0382688 | 1 | -1.82737 |
| Tcfl5 | 9.63562 | 3.68079 | 17.9239 | 5.6547 | 0.0164572 | 1 | -1.86017 |
| Ccdc68 | 31.1031 | 12.5429 | 58.8804 | 31.6007 | 0.041691 | 1 | -1.89307 |
| B230217O12Rik | 9.58672 | 7.22647 | 18.3013 | 3.29227 | 0.0177616 | 1 | -1.90903 |
| Mogat2 | 15.1296 | 4.77614 | 29.0264 | 7.42488 | 0.028655 | 1 | -1.91852 |
| Scml4 | 8.11044 | 4.43689 | 15.6758 | 6.85823 | 0.0435666 | 1 | -1.9328 |
| Dusp14 | 9.37651 | 2.36042 | 18.2048 | 4.34905 | 0.0339494 | 1 | -1.94153 |
| Wdr86 | 13.6745 | 6.76796 | 26.5935 | 14.3471 | 0.0425954 | 1 | -1.94475 |
| Doc2b | 10.1951 | 6.44158 | 19.9632 | 10.0368 | 0.0470372 | 1 | -1.95812 |
| Mpl | 9.62041 | 1.90811 | 19.5293 | 11.2748 | 0.0367953 | 1 | -2.02999 |
| Rerg | 34.4847 | 7.46566 | 71.2759 | 32.4632 | 0.0115577 | 1 | -2.06688 |
| Srrm4 | 5.54302 | 1.54903 | 11.6406 | 3.68979 | 0.00101424 | 0.501225 | -2.10005 |
| Cnnm1 | 11.9071 | 2.12002 | 25.7474 | 11.7418 | 0.018716 | 1 | -2.16236 |
| Actc1 | 13.6526 | 4.964 | 29.7696 | 11.9948 | 0.00459042 | 1 | -2.18052 |
| Adm2 | 14.7358 | 6.03752 | 33.0998 | 17.4742 | 0.0174222 | 1 | -2.24621 |
| C530005A16Rik | 7.42028 | 1.2102 | 16.7611 | 7.25969 | 0.0182233 | 1 | -2.25882 |
| H60b | 11.3557 | 6.2866 | 25.7718 | 11.2709 | 0.0149347 | 1 | -2.2695 |
| Slc6a2 | 6.01236 | 5.06147 | 13.7396 | 0.581872 | 0.00105065 | 0.501225 | -2.28523 |
| Gal | 7.45712 | 2.38859 | 17.1062 | 9.30445 | 0.0251976 | 1 | -2.29394 |
| Trim10 | 4.42432 | 3.05827 | 10.7568 | 5.79815 | 0.0398148 | 1 | -2.4313 |
| Rtkn2 | 14.9158 | 4.07486 | 37.1356 | 17.3316 | 0.0492667 | 1 | -2.48968 |
| Ankrd1 | 45.3955 | 28.2284 | 113.165 | 58.9734 | 0.0132232 | 1 | -2.49286 |
| Efna2 | 5.49688 | 1.12747 | 13.7782 | 4.72164 | 0.00676594 | 1 | -2.50656 |
| Gm9079 | 6.1463 | 5.48545 | 15.4248 | 7.53769 | 0.0177206 | 1 | -2.50961 |
| Fam181b | 8.36175 | 5.39405 | 21.6705 | 13.5423 | 0.02491 | 1 | -2.59162 |
| Mettl24 | 6.74893 | 3.05296 | 18.0959 | 12.6384 | 0.03356 | 1 | -2.68131 |
| Duoxa1 | 3.73914 | 1.04776 | 10.5234 | 4.55713 | 0.00416029 | 1 | -2.81439 |
| Gpx2-ps1 | 4.48446 | 1.72979 | 12.6227 | 4.66395 | 0.00789811 | 1 | -2.81478 |
| 1700007J10Rik | 4.96298 | 3.03281 | 14.4587 | 6.69074 | 0.0181612 | 1 | -2.9133 |
| D630003M21Rik | 4.67462 | 3.44364 | 13.7689 | 8.80941 | 0.0193447 | 1 | -2.94545 |
| Apon | 5.08962 | 3.54847 | 15.5373 | 9.55887 | 0.0182592 | 1 | -3.05274 |
| Cdnf | 3.44334 | 2.43553 | 11.7511 | 3.2618 | 0.00138488 | 0.565865 | -3.41271 |
| Gucy2f | 4.21035 | 2.12512 | 14.3858 | 10.87 | 0.0252984 | 1 | -3.41678 |
| Kcna6 | 6.77427 | 2.70062 | 25.2421 | 15.1418 | 0.00539066 | 1 | -3.72617 |
| Kcnd3 | 3.84042 | 2.46217 | 14.6867 | 9.03851 | 0.00674731 | 1 | -3.82423 |
| Gm3696 | 4.1341 | 6.2682 | 16.6323 | 13.6656 | 0.0366293 | 1 | -4.02319 |
| Tcf24 | 2.56836 | 2.21075 | 12.2361 | 4.31341 | 0.000273782 | 0.182756 | -4.76415 |
