# Supplementary material for: Tumor endothelial cell up-regulation of IDO1 is an immunosuppressive feed-back mechanism that reduces the response to CD40-stimulating immunotherapy
Source: Oncoimmunology. 2020 Mar 9;9(1):1730538. doi: 10.1080/2162402X.2020.1730538 (PMC7094447; doi:10.1080/2162402X.2020.1730538)
Supplement: Supplemental Material [file koni-09-01-1730538-s001.zip › Table_S4_Word_File latest.docx]

| Table S4: GSEA for B16.F10-derived TECs (anti-CD40 vs isotype) | | | | | | |
| --- | --- | --- | --- | --- | --- | --- |
| Gene Set | Number of Markers | ES | NES | p-value | FDR | Description |
| HALLMARK_INTERFERON_GAMMA_RESPONSE | 163 | 0.775018 | 1.77745 | 0.0117807 | 0.0743337 | TEC_anti-CD40 up vs TEC_isotype |
| HALLMARK_IL6_JAK_STAT3_SIGNALING | 69 | 0.605254 | 1.59857 | 0.0198567 | 0.0889787 | TEC_anti-CD40 up vs TEC_isotype |
| HALLMARK_INTERFERON_ALPHA_RESPONSE | 75 | 0.788965 | 1.64788 | 0.0312719 | 0.0944085 | TEC_anti-CD40 up vs TEC_isotype |
| HALLMARK_XENOBIOTIC_METABOLISM | 141 | 0.329532 | 1.25339 | 0.132801 | 0.766529 | TEC_anti-CD40 up vs TEC_isotype |
| HALLMARK_OXIDATIVE_PHOSPHORYLATION | 171 | -0.595876 | -1.42683 | 0.145916 | 1 | TEC_anti-CD40 down vs TEC_isotype |
| HALLMARK_INFLAMMATORY_RESPONSE | 174 | 0.518626 | 1.4194 | 0.157004 | 0.429111 | TEC_anti-CD40 up vs TEC_isotype |
| HALLMARK_UNFOLDED_PROTEIN_RESPONSE | 107 | -0.44504 | -1.21232 | 0.191807 | 1 | TEC_anti-CD40 down vs TEC_isotype |
| HALLMARK_MTORC1_SIGNALING | 180 | -0.488048 | -1.2461 | 0.200623 | 1 | TEC_anti-CD40 down vs TEC_isotype |
| HALLMARK_WNT_BETA_CATENIN_SIGNALING | 36 | 0.456856 | 1.20945 | 0.203304 | 0.797564 | TEC_anti-CD40 up vs TEC_isotype |
| HALLMARK_BILE_ACID_METABOLISM | 82 | 0.37031 | 1.16482 | 0.207886 | 0.614484 | TEC_anti-CD40 up vs TEC_isotype |
| HALLMARK_COMPLEMENT | 148 | 0.422783 | 1.1942 | 0.229006 | 0.744929 | TEC_anti-CD40 up vs TEC_isotype |
| HALLMARK_ESTROGEN_RESPONSE_EARLY | 157 | 0.367106 | 1.16867 | 0.233884 | 0.671688 | TEC_anti-CD40 up vs TEC_isotype |
| HALLMARK_TNFA_SIGNALING_VIA_NFKB | 181 | 0.523906 | 1.31253 | 0.235624 | 0.666985 | TEC_anti-CD40 up vs TEC_isotype |
| HALLMARK_APOPTOSIS | 143 | 0.389958 | 1.17463 | 0.253355 | 0.728297 | TEC_anti-CD40 up vs TEC_isotype |
| HALLMARK_MYC_TARGETS_V1 | 168 | -0.597911 | -1.21781 | 0.27172 | 1 | TEC_anti-CD40 down vs TEC_isotype |
| HALLMARK_KRAS_SIGNALING_DN | 89 | -0.356666 | -1.085 | 0.284322 | 1 | TEC_anti-CD40 down vs TEC_isotype |
| HALLMARK_P53_PATHWAY | 176 | 0.340391 | 1.13121 | 0.293735 | 0.664606 | TEC_anti-CD40 up vs TEC_isotype |
| HALLMARK_CHOLESTEROL_HOMEOSTASIS | 66 | -0.372961 | -1.09127 | 0.363806 | 1 | TEC_anti-CD40 down vs TEC_isotype |
| HALLMARK_ADIPOGENESIS | 177 | -0.326163 | -1.01256 | 0.364971 | 0.821735 | TEC_anti-CD40 down vs TEC_isotype |
| HALLMARK_HEME_METABOLISM | 159 | 0.317943 | 1.1151 | 0.395573 | 0.646917 | TEC_anti-CD40 up vs TEC_isotype |
| HALLMARK_FATTY_ACID_METABOLISM | 124 | -0.372803 | -1.0353 | 0.400537 | 1 | TEC_anti-CD40 down vs TEC_isotype |
| HALLMARK_PROTEIN_SECRETION | 88 | -0.437218 | -1.08055 | 0.416815 | 0.955988 | TEC_anti-CD40 down vs TEC_isotype |
| HALLMARK_GLYCOLYSIS | 162 | -0.325472 | -0.9733 | 0.424888 | 0.78648 | TEC_anti-CD40 down vs TEC_isotype |
| HALLMARK_ANDROGEN_RESPONSE | 88 | -0.425772 | -1.09158 | 0.425737 | 1 | TEC_anti-CD40 down vs TEC_isotype |
| HALLMARK_DNA_REPAIR | 132 | -0.409865 | -1.08268 | 0.44121 | 1 | TEC_anti-CD40 down vs TEC_isotype |
| HALLMARK_G2M_CHECKPOINT | 184 | -0.423147 | -1.02821 | 0.441844 | 0.926713 | TEC_anti-CD40 down vs TEC_isotype |
| HALLMARK_ESTROGEN_RESPONSE_LATE | 153 | 0.285669 | 0.996812 | 0.450609 | 0.681718 | TEC_anti-CD40 up vs TEC_isotype |
| HALLMARK_PEROXISOME | 79 | 0.315801 | 1.00223 | 0.46875 | 0.713755 | TEC_anti-CD40 up vs TEC_isotype |
| HALLMARK_TGF_BETA_SIGNALING | 48 | 0.431575 | 1.02295 | 0.481976 | 0.752362 | TEC_anti-CD40 up vs TEC_isotype |
| HALLMARK_IL2_STAT5_SIGNALING | 170 | 0.321236 | 1.04666 | 0.506812 | 0.749204 | TEC_anti-CD40 up vs TEC_isotype |
| HALLMARK_E2F_TARGETS | 184 | -0.491204 | -1.02771 | 0.506867 | 0.852899 | TEC_anti-CD40 down vs TEC_isotype |
| HALLMARK_APICAL_SURFACE | 32 | -0.355745 | -0.98058 | 0.519839 | 0.830902 | TEC_anti-CD40 down vs TEC_isotype |
| HALLMARK_COAGULATION | 96 | 0.396689 | 1.01889 | 0.54961 | 0.717061 | TEC_anti-CD40 up vs TEC_isotype |
| HALLMARK_UV_RESPONSE_DN | 134 | 0.362948 | 0.904687 | 0.621065 | 0.812068 | TEC_anti-CD40 up vs TEC_isotype |
| HALLMARK_KRAS_SIGNALING_UP | 157 | 0.335157 | 0.862309 | 0.626781 | 0.848569 | TEC_anti-CD40 up vs TEC_isotype |
| HALLMARK_UV_RESPONSE_UP | 125 | -0.26878 | -0.85885 | 0.643485 | 0.970148 | TEC_anti-CD40 down vs TEC_isotype |
| HALLMARK_SPERMATOGENESIS | 74 | 0.277913 | 0.85717 | 0.659123 | 0.816819 | TEC_anti-CD40 up vs TEC_isotype |
| HALLMARK_HEDGEHOG_SIGNALING | 31 | -0.315681 | -0.82439 | 0.663629 | 0.973206 | TEC_anti-CD40 down vs TEC_isotype |
| HALLMARK_ANGIOGENESIS | 30 | 0.368256 | 0.786438 | 0.686036 | 0.843891 | TEC_anti-CD40 up vs TEC_isotype |
| HALLMARK_EPITHELIAL_MESENCHYMAL_TRANSITION | 175 | -0.364458 | -0.76937 | 0.710024 | 1 | TEC_anti-CD40 down vs TEC_isotype |
| HALLMARK_NOTCH_SIGNALING | 31 | 0.300767 | 0.823266 | 0.712253 | 0.827724 | TEC_anti-CD40 up vs TEC_isotype |
| HALLMARK_REACTIVE_OXIGEN_SPECIES_PATHWAY | 40 | -0.290142 | -0.73701 | 0.733677 | 0.846619 | TEC_anti-CD40 down vs TEC_isotype |
| HALLMARK_PANCREAS_BETA_CELLS | 15 | -0.355512 | -0.74389 | 0.736908 | 0.957672 | TEC_anti-CD40 down vs TEC_isotype |
| HALLMARK_MYC_TARGETS_V2 | 54 | -0.356216 | -0.74123 | 0.745417 | 0.917935 | TEC_anti-CD40 down vs TEC_isotype |
| HALLMARK_PI3K_AKT_MTOR_SIGNALING | 93 | -0.25943 | -0.76404 | 0.803312 | 0.975861 | TEC_anti-CD40 down vs TEC_isotype |
| HALLMARK_MITOTIC_SPINDLE | 187 | -0.288462 | -0.74114 | 0.81 | 0.877135 | TEC_anti-CD40 down vs TEC_isotype |
| HALLMARK_MYOGENESIS | 161 | 0.274377 | 0.749125 | 0.829382 | 0.855808 | TEC_anti-CD40 up vs TEC_isotype |
| HALLMARK_HYPOXIA | 165 | 0.256897 | 0.662367 | 0.893223 | 0.903403 | TEC_anti-CD40 up vs TEC_isotype |
| HALLMARK_APICAL_JUNCTION | 152 | -0.260558 | -0.61832 | 0.945776 | 0.921415 | TEC_anti-CD40 down vs TEC_isotype |
| HALLMARK_ALLOGRAFT_REJECTION | 145 | 0 | ? | 1 | 1 | No change |
