# Supplementary figures and images for: Tumor endothelial cell up-regulation of IDO1 is an immunosuppressive feed-back mechanism that reduces the response to CD40-stimulating immunotherapy
Source: Oncoimmunology. 2020 Mar 9;9(1):1730538. doi: 10.1080/2162402X.2020.1730538 (PMC7094447; doi:10.1080/2162402X.2020.1730538)

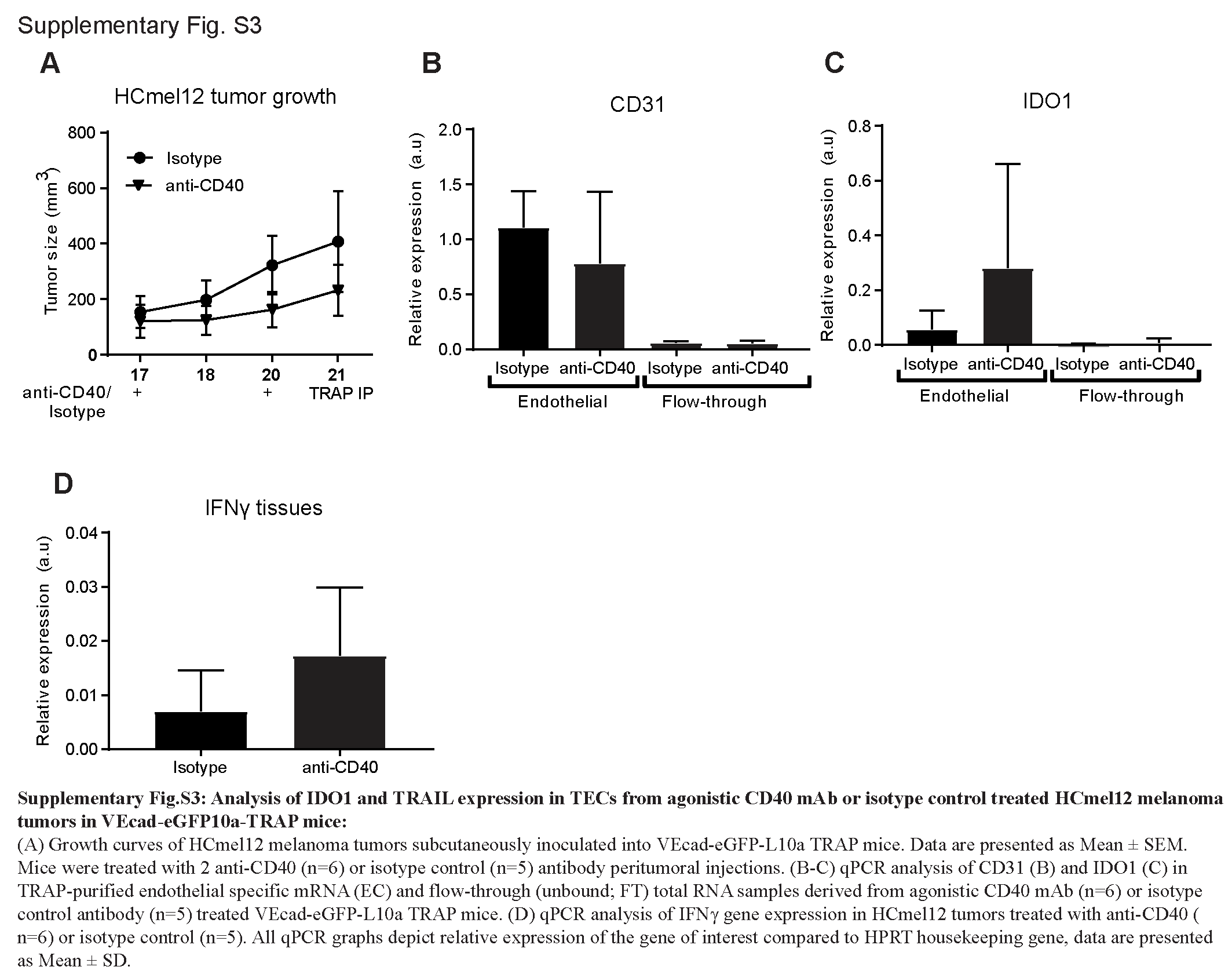

Supplement: Supplemental Material [file koni-09-01-1730538-s001.zip › 20171017_sup.fig_3.tif]

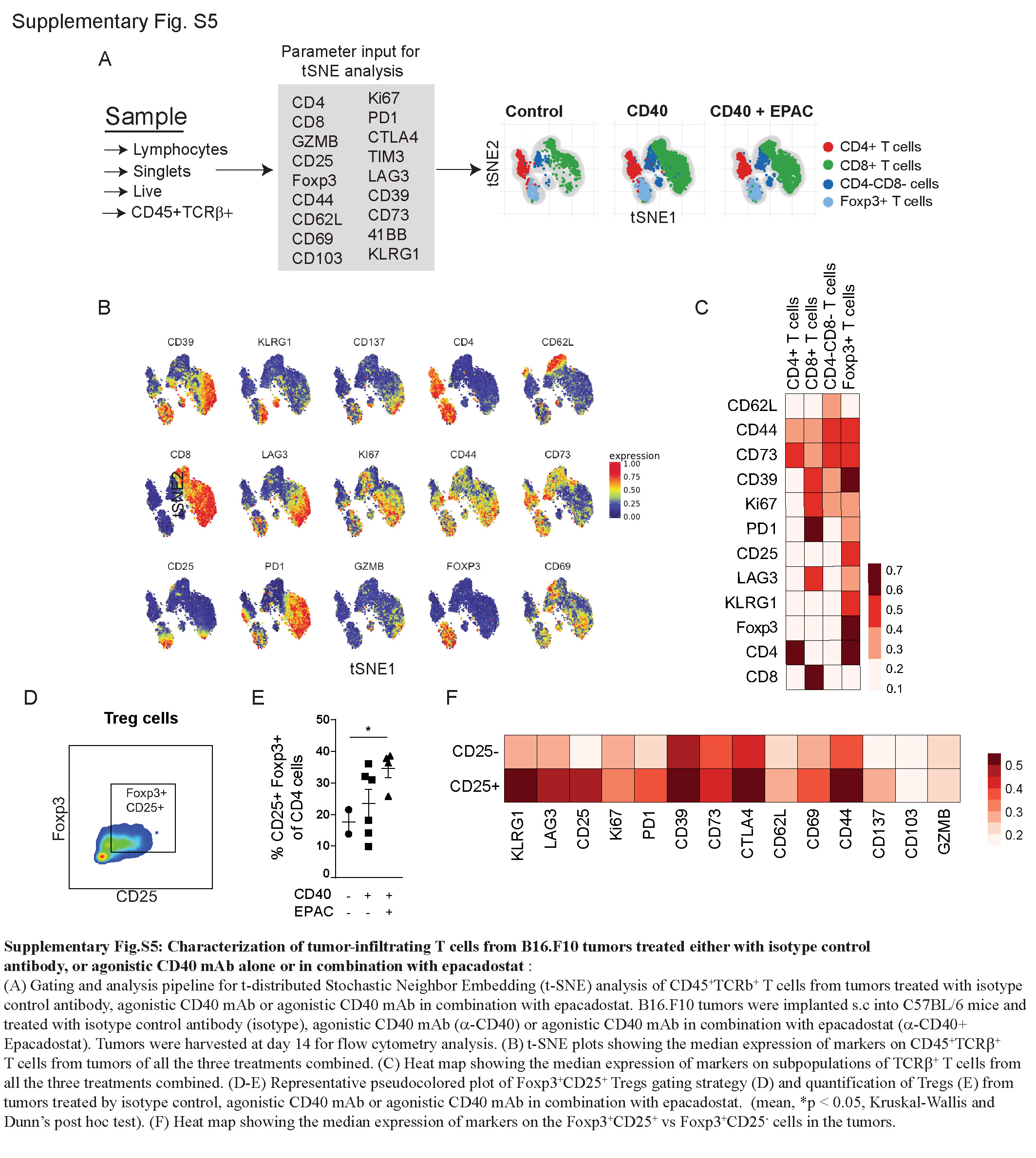

Supplement: Supplemental Material [file koni-09-01-1730538-s001.zip › 20171017_sup.fig_5.tif]

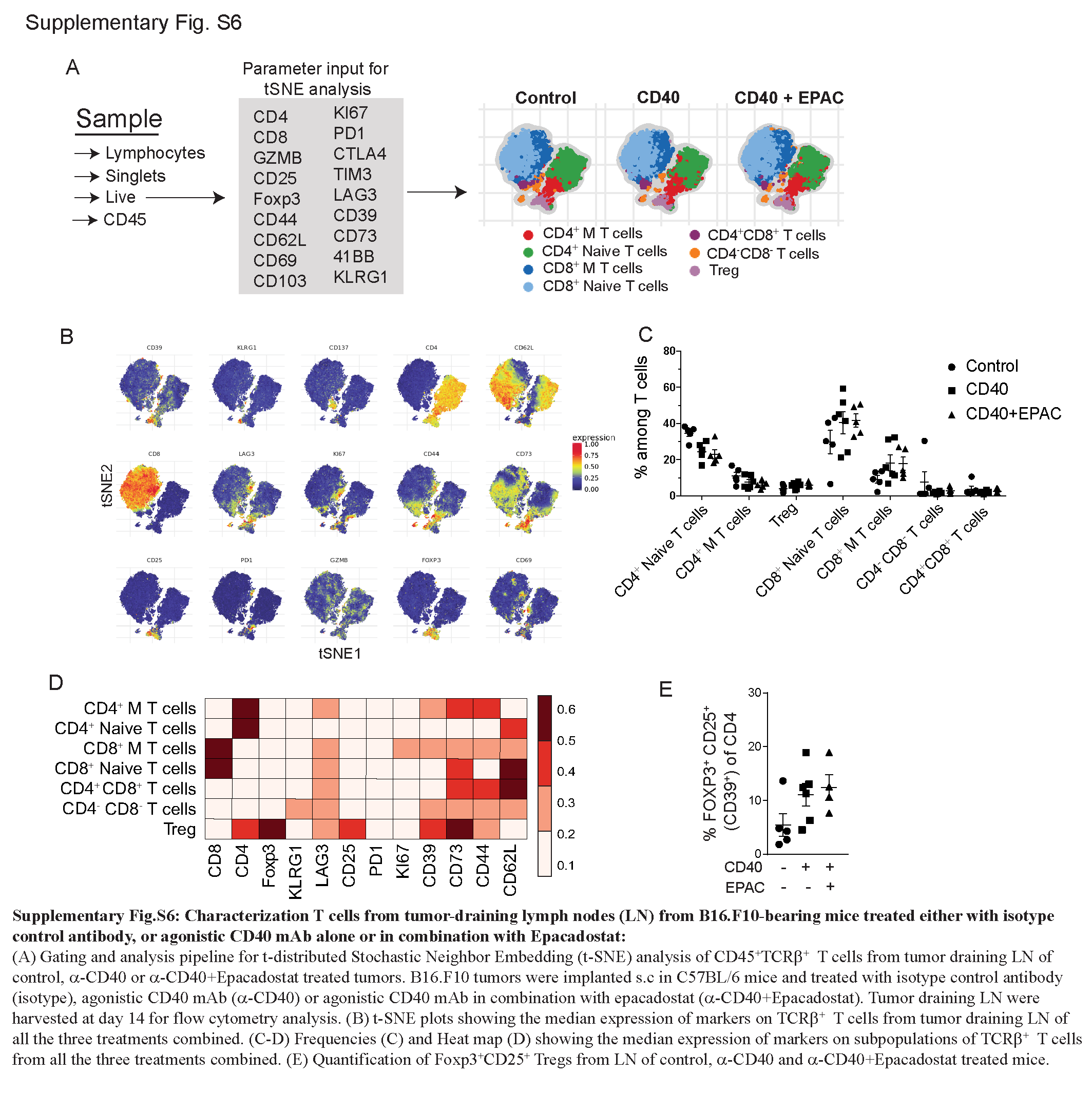

Supplement: Supplemental Material [file koni-09-01-1730538-s001.zip › 20171017_sup.fig_6.tif]

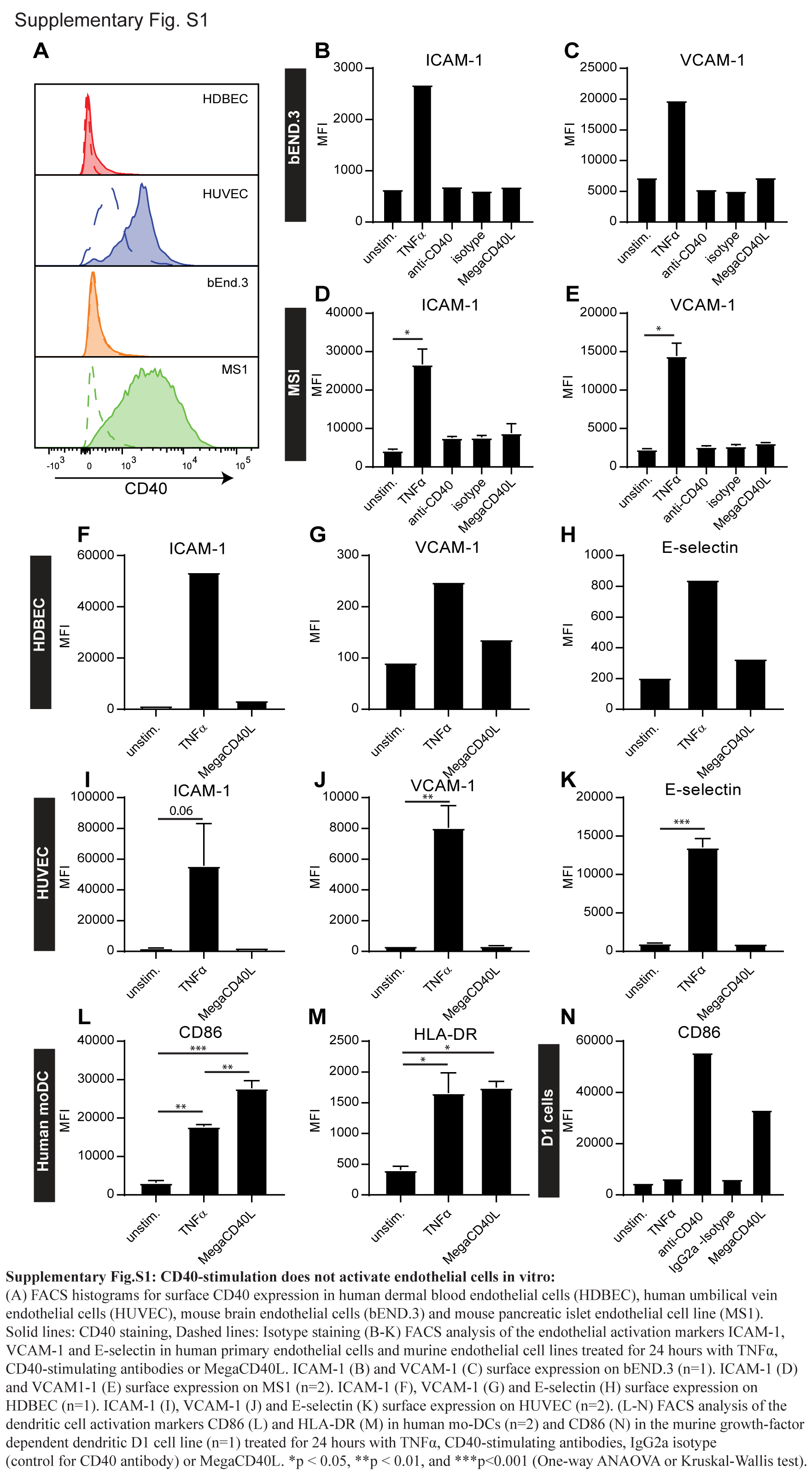

Supplement: Supplemental Material [file koni-09-01-1730538-s001.zip › sup.fig_01.tif]

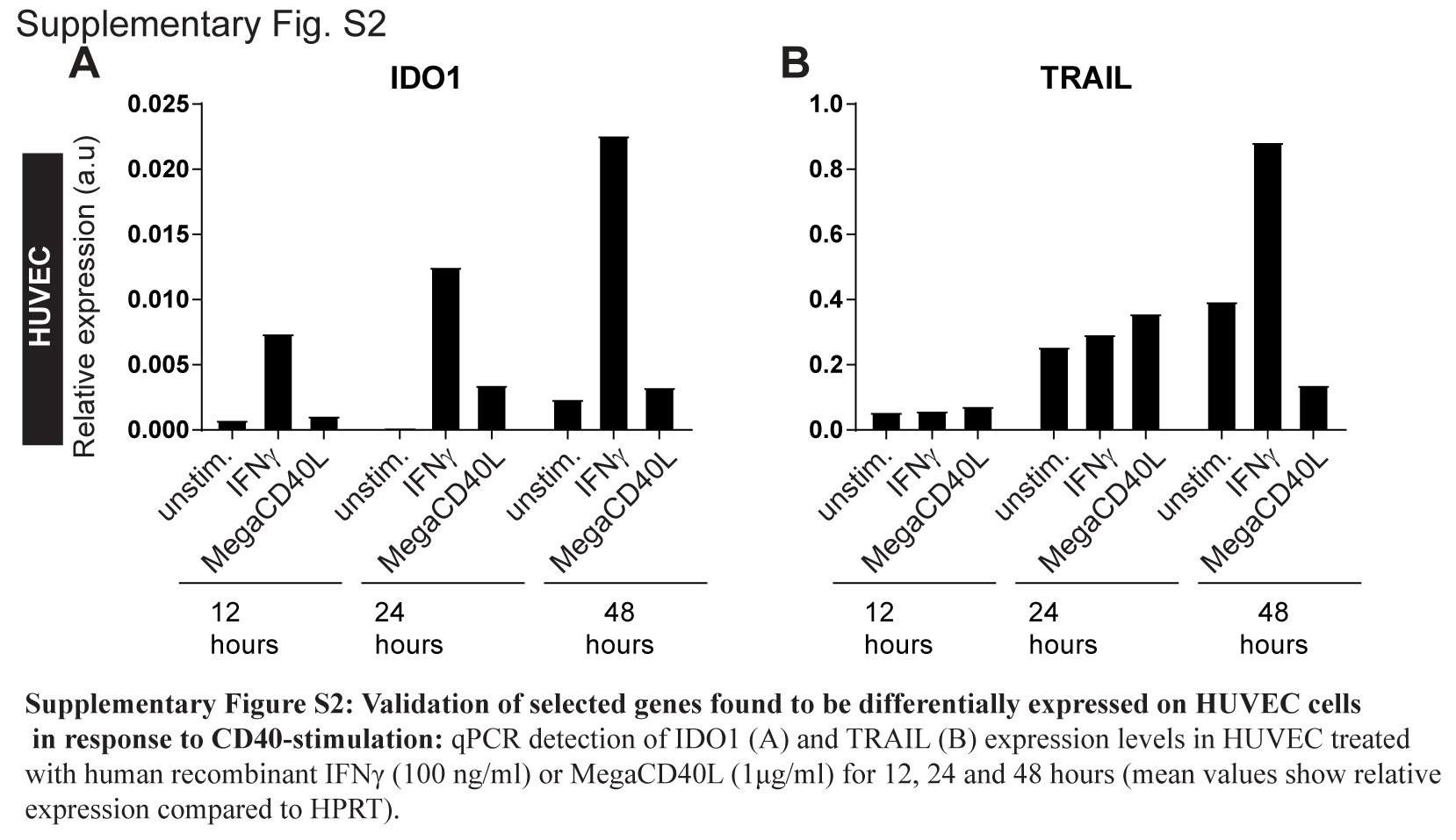

Supplement: Supplemental Material [file koni-09-01-1730538-s001.zip › sup.fig_02.tif]

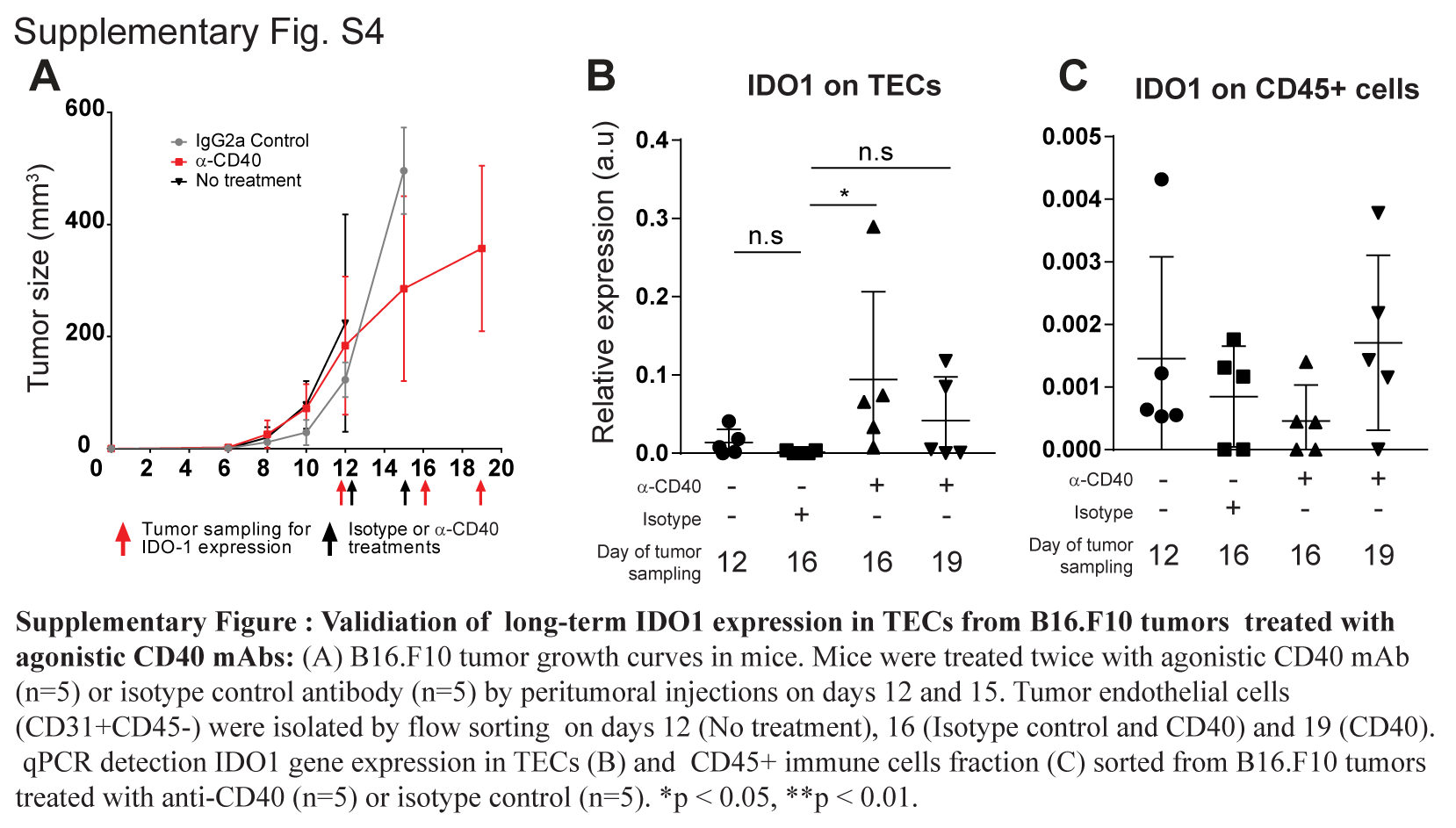

Supplement: Supplemental Material [file koni-09-01-1730538-s001.zip › sup.fig_04.tif]
